# Modularity Induced Gating and Delays in Neuronal Networks

**DOI:** 10.1371/journal.pcbi.1004883

**Published:** 2016-04-22

**Authors:** Mark Shein-Idelson, Gilad Cohen, Eshel Ben-Jacob, Yael Hanein

**Affiliations:** 1 School of Electrical Engineering, Tel Aviv University, Tel Aviv, Israel; 2 Tel Aviv University Center for Nanoscience and Nanotechnology, Tel Aviv, Israel; 3 Max Planck Institute for Brain Research, Frankfurt am Main, Germany; 4 School of Physics and Astronomy, Tel Aviv University, Tel Aviv, Israel; 5 Sagol School of Neuroscience, Tel Aviv University, Tel Aviv, Israel; Université Paris Descartes, Centre National de la Recherche Scientifique, FRANCE

## Abstract

Neural networks, despite their highly interconnected nature, exhibit distinctly localized and gated activation. Modularity, a distinctive feature of neural networks, has been recently proposed as an important parameter determining the manner by which networks support activity propagation. Here we use an engineered biological model, consisting of engineered rat cortical neurons, to study the role of modular topology in gating the activity between cell populations. We show that pairs of connected modules support conditional propagation (transmitting stronger bursts with higher probability), long delays and propagation asymmetry. Moreover, large modular networks manifest diverse patterns of both local and global activation. Blocking inhibition decreased activity diversity and replaced it with highly consistent transmission patterns. By independently controlling modularity and disinhibition, experimentally and in a model, we pose that modular topology is an important parameter affecting activation localization and is instrumental for population-level gating by disinhibition.

## Author Summary

The capacity to transmit information between connected parts of a neuronal network is fundamental to its function. The organization of network connections (the topology of the network) is therefore expected to play an important role in determining network transmission. Since modular topology characterizes many brain circuits on multiple scales, investigating the role of modularity in activity gating is clearly desirable. By engineering such modular networks in vitro, we were able to perform such an investigation. Under these experimental conditions, we can independently control the degree of modularity, as well as inhibition in the network. We show that a combination of these two properties is highly beneficial from a communication perspective. Namely, it equips connected modules and large modular networks with the capacity to gate and temporally coordinate activity between the different parts of the network.

## Introduction

Activity gating and control over propagation are fundamental capacities of neural circuits. It is widely accepted that population-level gating is strongly affected by changing the balance between excitation and inhibition in connected sub-populations of neurons [1–6]. However, while the role of excitation-inhibition has been widely investigated, the contribution of circuit topology to activity gating has received much less attention. Modular topology is of particular interest, as it is a fundamental feature of biological neuronal circuits [7–11]. Modular circuits are composed of highly connected groups of neurons (modules) which are loosely connected to other groups. Such a network organization is found at many spatial scales, ranging from anatomically defined brain regions to groups of neurons [7,8,12,13].

Experimental investigation into the contribution of modular topology to gating phenomena is faced with major challenges. While large brain areas have well-documented connectivity and activity maps [12,14,15], accessing complete brain circuits at smaller scales is still limited. Foremost, the connectivity maps are highly untraceable in the three-dimensional architecture of the tissue. In addition, since cell assemblies are often dispersed in space, their simultaneous identification and recording are still beyond the reach of contemporary technologies [16]. Finally, as neuronal circuits are not prone to design, systematic studies are impossible. Consequently, studies aimed to relate activity propagation and gating to network architecture (such as modular networks) are mainly restricted to theoretical investigations [17].

Indeed, theoretical studies indicate that modular organization greatly impacts network functionality [18]. Modular circuits provide control over activity propagation [9,10,19,20], time-scale separation [21], dynamical complexity and the computational capacity of the network [10,22–25].

In this study we experimentally address, for the first time, the relation between circuit modularity and activity gating. To overcome the inherent limitations associated with studying intact tissues, we utilized a cell patterning technique to induce self-organization of modular networks in culture. We found that pairs of connected modules support conditional propagation that is dependent on the activity intensity in the sending module. In large networks of many connected modules, conditional propagation enhances the diversity of activation patterns, and is manifested as events initiated at different modules which then propagate to different distances. Interestingly, blocking network inhibition decreased the activity diversity and replaced the conditional propagation with highly reliable transmission. These features are absent in the activity repertoire of uniform networks. Thus, we show how a combination of modular circuit architecture and disinhibition supports gating.

## Results

For the sake of clarity, the results are organized according to systems with growing complexity. We begin by addressing the properties of connected cluster pairs. We then address chains of more than two clusters. Next, we look at the activation repertoires of larger systems (networks of connected clusters). Finally, we attempt to explain our results with a computational model addressing the accumulative effects of disinhibition and modular topology.

### Engineering modular networks in vitro

**Fig 1:**
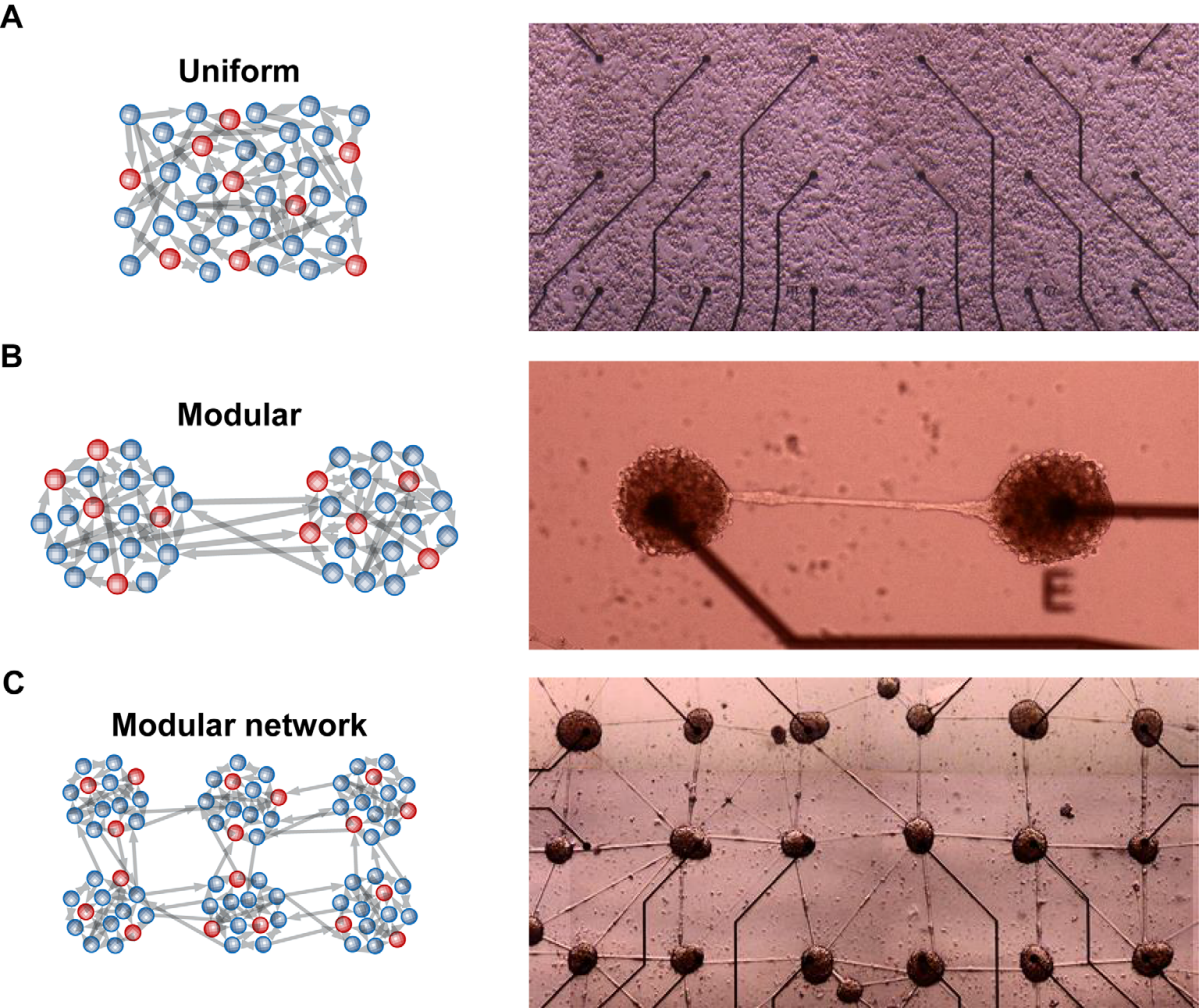
Uniform versus engineered circuits in culture (left–schematics, right–in vitro model). (A) Uniform network. Cells (inhibitory, red, and excitatory, blue) are uniformly distributed and their probability to connect to other neurons is the same. (B) A simple, two cluster modular network. Neurons are divided into two distinct cell populations. (C) A large network of many connected modules. Electrode-electrode (center to center) distance is 500 μm.

To investigate how activity propagates through modular neuronal networks, we used a unique biological model system of engineered clusters in vitro. Control over circuit architecture was achieved using heterogeneous surfaces with different degrees of adhesiveness (see Materials and Methods). Specifically, islands of highly adhesive surfaces were realized on a non-adhesive background. Due to their innate propensity to cluster, neurons self-organized into modular circuits within several days in culture (Fig 1), in accordance with previous work [26–28]. Each of the clusters comprised of several tens to hundreds of neurons (S1A Fig), connected through a bundle of fasciculated neurites (Fig 1). The number of cells per cluster was estimated from the cluster area using N = 0.0079S-1.9, where N is the number of cells and S is the cluster area in μm^2^. This relation was calculated in a previous publication in which clusters were grown under the same experimental conditions [29]. In contrast to previous methods [30–33], our procedure allows the formation of small-scale sub-networks of different sizes, ranging from cluster chains (Fig 1B) to two-dimensional networks of many connected clusters (Fig 1C). The position of each cluster was aligned with a micro electrode, allowing local electrical recording from each cluster (Fig 1B and 1C-right).

### Synchronized bursts in modular networks

**Fig 2:**
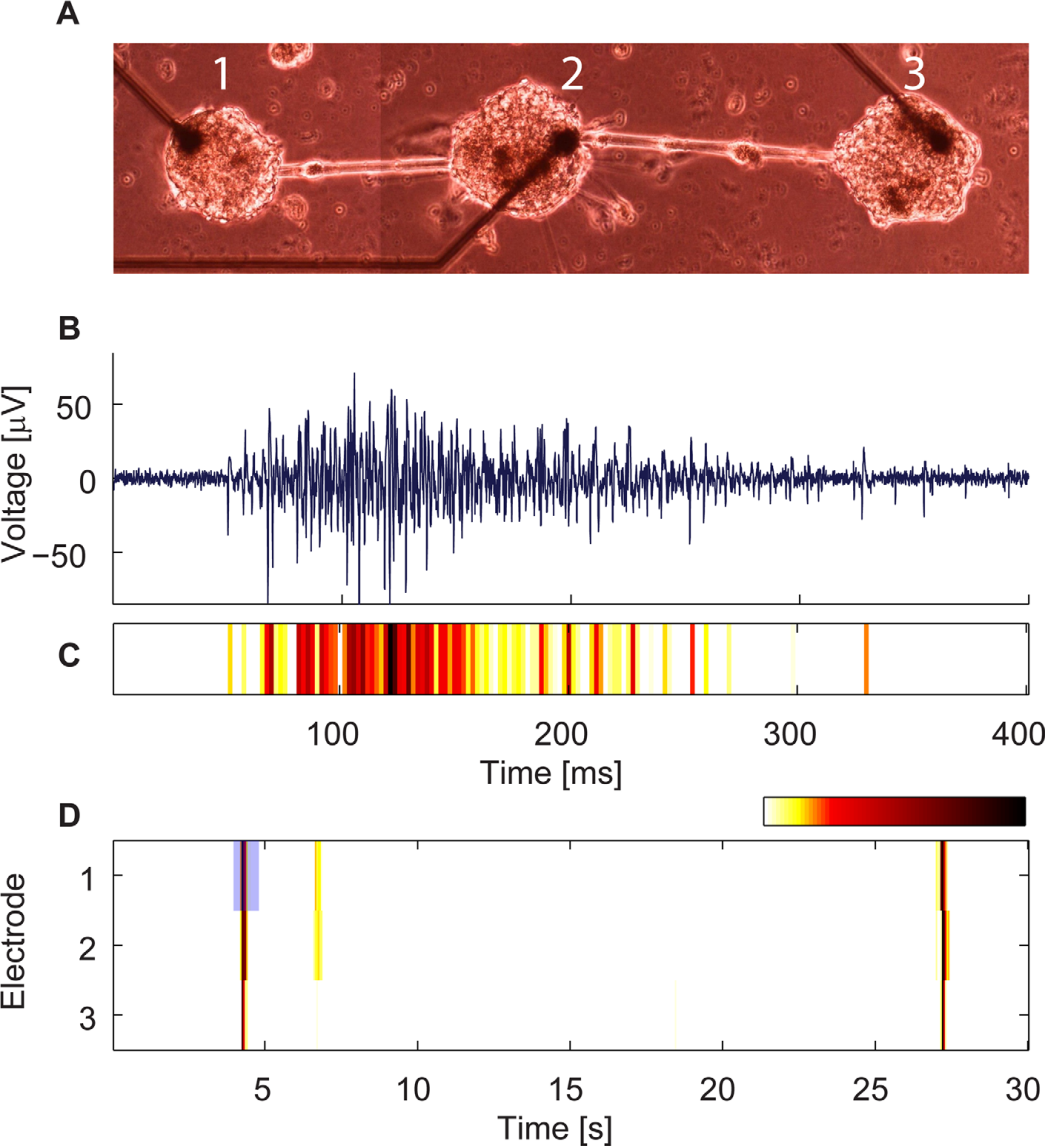
Recordings from a modular network in culture. (A) A bright field image of a three cluster chain. (B) A voltage trace recorded from electrode 1 in (A) during a network burst. (C) The voltage trace from (B) is represented by the activity intensity (see Materials and Methods) and is shown in a color code. (D) Activity intensity raster plot recorded from the network in (A). The blue rectangle marks the network burst in (C). Electrode-electrode (center to center) distance is 500 μm.

Using the embedded electrodes (Fig 2A), the simultaneous activity of all clusters was recorded. The vast majority of measured clusters were found to be spontaneously active after several days in culture. A typical voltage trace recorded from one electrode is shown in Fig 2B. Each electrode recorded the superimposed activity of many neurons within each cluster. It was previously shown that voltage traces represent spike summation and correspond to the increase and decrease in population activity [29,34]. Accordingly, to represent the activity intensity of such clusters, we averaged the rectified voltage traces over short time windows and color coded them (Fig 2C) (see Materials and Methods for details). Spontaneous activity of individual clusters was characterized by typical features previously observed in developing networks [29,35,36]. We observed the activation of network bursts (NBs) which are short epochs of network intense firing, separated by longer periods of sporadic activity (Fig 2D). To verify that these events represent the collective activity of many neurons that synchronize within the NB time window, we performed recordings from single clusters using dense electrode arrays (30 μm spacing) with a smaller electrode surface area (314 μm^2^ in contrast to 2827 μm^2^ in our regular electrodes) which pick up activity from more local populations of neurons. The variability in the spiking profiles across different electrodes exemplified the synchronized nature of spiking within NBs of single clusters (S2 Fig). While the activity within clusters appeared to be synchronized, between connected clusters the synchrony was transient.

### Conditional propagation

**Fig 3:**
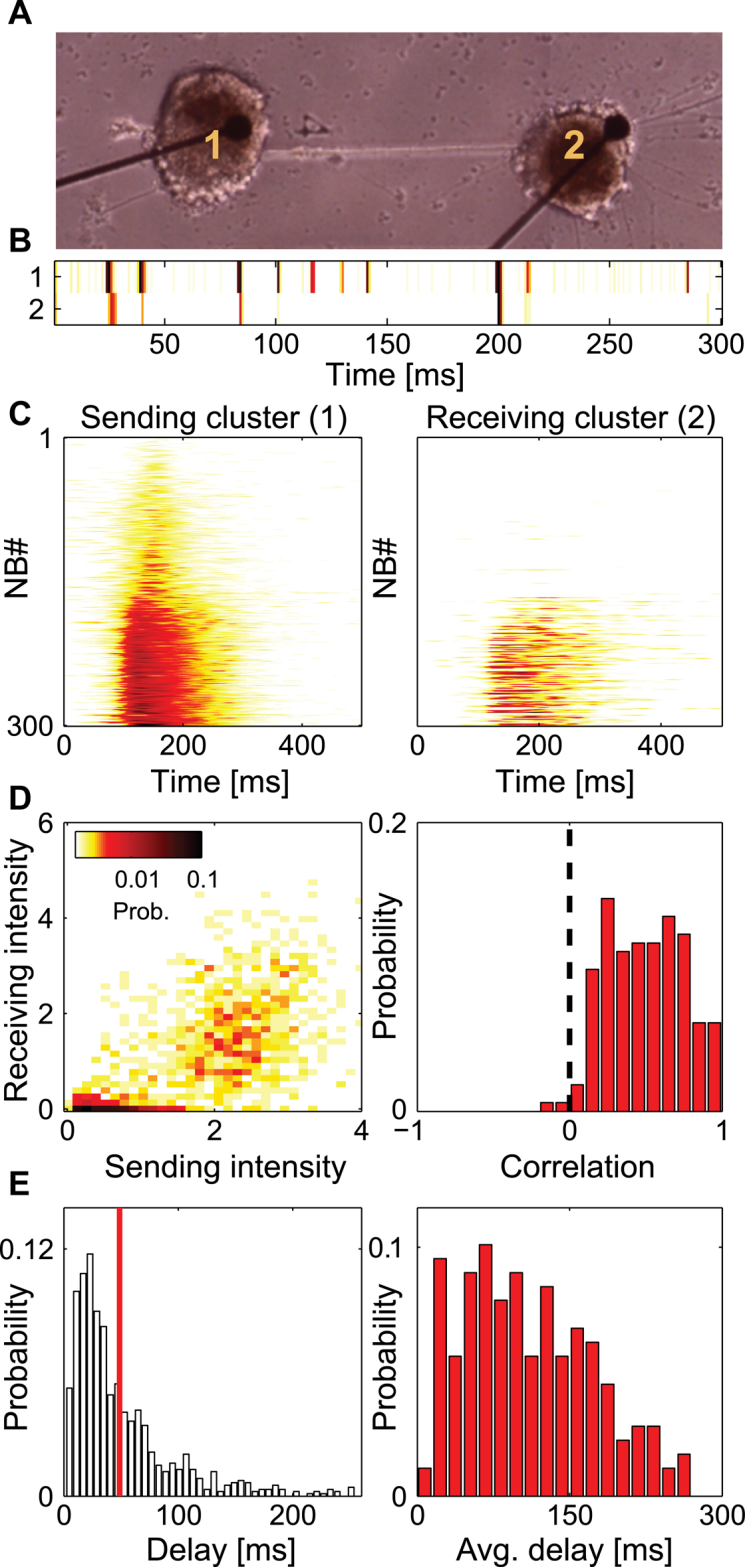
Conditional propagation between clusters. (A) A Bright field image of a cluster pair (500 μm distance between electrode centers). (B) Activity intensity traces recorded from the two clusters in (A) (color code as in Fig 2). (C) 300 consecutive smoothed activity intensity traces recorded during NBs, detected in cluster 1 (left) and their responses as recorded from cluster 2 (right). NBs propagating from cluster 2 to 1 are not shown. Traces were rearranged according to increasing total NB activity intensity. High intensity NBs propagated to the receiving cluster at much greater probability. (D-left) Color coded probability of the NB intensity in the receiving cluster as a function of the NB intensity in the sending cluster (calculated for 1634 consecutive NBs). (D-right) The correlation between the NB intensity in the receiving and sending clusters for different cluster pairs. 163 pairs were included in the analysis. 15 pairs had a correlation with a P-Value (t-test) higher than 0.005 and were excluded. (E-left) Delay distribution between the sending and receiving clusters for the cluster pair in (A). The average delay is marked by a red line. (E-right) Distribution of the average delays of 178 cluster pairs. The average delays were in the order of tens of milliseconds and reached values as high as 200 ms.

We begin by examining the activity of connected cluster pairs (Fig 3A). NBs were found to be either confined to a single cluster or spread over nearby connecting clusters (Fig 3B). To investigate whether the activity propagation between connected clusters was related to the activity intensity in the clusters, we examined the propagation between pairs of adjacent clusters. For each pair, we defined one cluster as the "sending cluster" and detected all NBs occurring in this cluster (see details in Materials and Methods). From this NB pool, we selected only NBs which were activated in the sending cluster before the neighboring cluster, defined here as the "receiving cluster". This selection process consisted of rejecting NBs according to the delay calculated from the peak position of the cross-correlation function of the smoothed (convoluted with a Gaussian kernel, σ = 10ms) activity intensities of the sending and receiving clusters. Positive offset delay was attributed to propagation from a receiving to a sending cluster. Fig 3A–3C illustrates activity propagation in a representative cluster pair. 300 consecutive NB traces, recorded from cluster 1 (the sending cluster) in Fig 3A, are shown in Fig 3C-left. These NBs were reordered according to increased NB intensity (sum over the NB activity intensity trace) in the sending cluster. The receiving cluster responses to these NBs are shown in Fig 3C-right. The total intensity of these responses (averaged over all NBs) were only slightly lower than in the sending cluster (Z-score of the difference in AI was 0.083), but significant (PV = 0.013, Mann-Whitney-Wilcoxon test). Low intensity NBs did not propagate to the receiving cluster, while strong NBs did. It should be noted, however, that a small fraction of strong NBs failed to propagate. We termed this selective activation of NBs in the receiving cluster following an NB in the sending cluster as conditional propagation.

To quantify this behavior over many cluster pairs, we calculated for each NB in the selected pool, and for each cluster pair, the probability of having different normalized NB intensities (intensities divided by the intensity standard deviation) in the receiving cluster as a function of normalized NB intensities in the sending cluster. Data of the cluster pair in Fig 3A are presented in Fig 3D-left. This representation further illustrates that low intensity NBs did not yield strong responses in the receiving cluster, while NBs stronger than a certain value successfully propagated to the receiving cluster. It is important to note that in the example shown here, a distinct threshold between non-propagating and propagating NBs is apparent (Fig 3D-left). To quantify this threshold-like behavior, we calculated the bi-modality measure [37] on the distribution of normalized NB intensities projected on the identity line (S4A Fig) for the cluster pair in Fig 3D-left (S4B Fig), and for all cluster pairs (S4C Fig). Most clusters showed values larger than (0.555) corresponding to a tendency to bi-modality over uni-modality (S4C Fig). We verified these results by performing visual inspection of the distributions and determined that nearly half (48%) of the pairs showed a clear intensity threshold in the propagation probability. However, taking into account also pairs that did not show strong bi-modality, the general rule was that strong activations in the sending cluster yielded strong responses in the receiving cluster and vice versa. This effect was quantified by calculating the correlation between the normalized NB intensity of the receiving and sending clusters for all NBs in 163 cluster pairs, from 26 cultures (Fig 3D-right). Two types of cluster networks were considered in the analysis: The first type is one dimensional chain of clusters and the second involves a cluster chain with one of the clusters connected to a larger network (of many connected clusters). Within these networks, only pairs connected exclusively through a neurite bundle (and not through any other pathway) were analyzed. The data in Fig 3D-right exhibits a clear preference towards positive values, further suggesting that information about firing intensity is utilized by neuronal networks to control propagation between connected sub-populations. No significant correlation was observed between NB intensity correlation strength and the normalized difference in cluster cell numbers (C = 0.084, PV = 0.28).

### Sparse connectivity between sub-populations increases propagation delays

We next sought to investigate how modularity affects propagation delays. Delays in our engineered networks were evaluated by extracting the location of the peak in the cross-correlation function of every pair and every NB (as previously described). Only NBs propagating from the sending cluster to the receiving cluster were considered. The delay distribution across all NBs is shown in Fig 3E-left. The average delay was several tens of milliseconds (red line in Fig 3E-left) and the maximal value reached was 250 ms. With a distance between clusters of 500 μm, a delay of 100 ms corresponds to a propagation speed of 5 μm/ms. Long delays were also observed in other cluster pairs, as shown by the distribution of average delays (Fig 3E-right). We did not find a significant correlation (C = 0.060, PV = 0.43) between the average delay and the normalized absolute difference in cell numbers ($\frac{\left|N_{1}{-N}_{2}\right|}{{2(N}_{1}+N_{2})}$, N_i_ being the number of cells in each cluster). Interestingly, a weak positive correlation was found between average delay and the number of cells in the receiving cluster (C = 0.202, PV = 0.007)(S1C Fig), but not in the sending cluster (C = 0.098, PV = 0.19), suggesting that the delay is associated with the network recruitment time in the receiving cluster. Correspondingly, the average recruitment time for all NBs in a cluster, measured as the time between NB onset and NB peak (see NB detection in Materials and Methods), had similar time scales to the observed average delays (S3 Fig).

### Modularity supports asymmetric activity propagation

**Fig 4:**
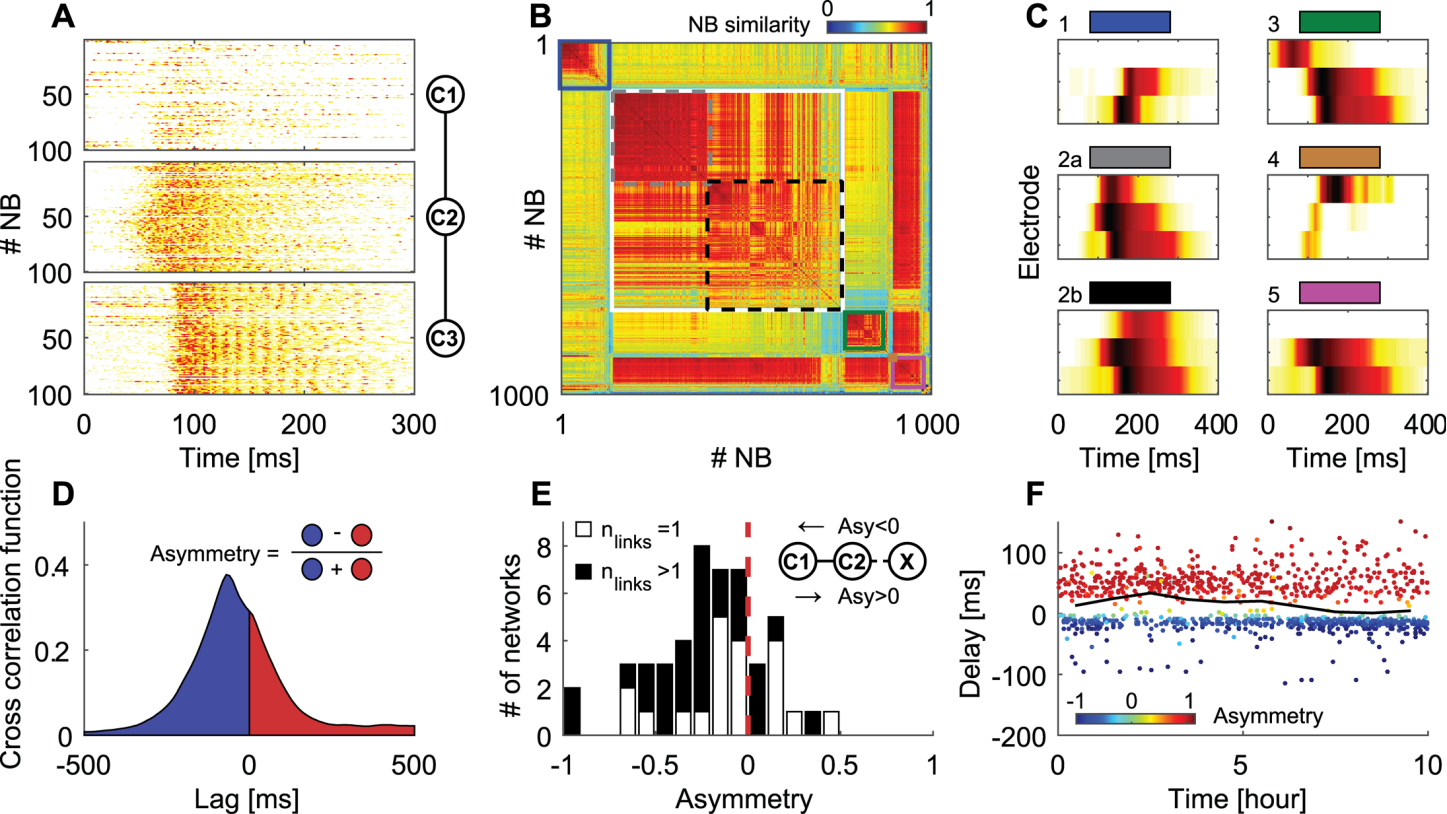
Asymmetry in modular networks. (A) Activity intensity of 100 consecutive NBs for each of the clusters in a three cluster chain. Inset: a schematic drawing of the network under investigation. (B) Reordered similarity matrix (see Materials and Methods for details) between 1000 consecutive NBs in this cluster chain. Only NBs in more than one cluster were considered. A clear separation of NB patterns into similarity groups is seen. (C) Average NB activity patterns are marked by the solid line rectangles in (B). Pattern number 2 was divided into two groups (2a and 2b, dashed lines) to exemplify variability within a group. (D) A schematic representation of the functional asymmetry calculation. Long-term asymmetry is calculated from the cross-correlations function as the integral over its positive side subtracted from its negative side and divided by the sum of both. (E) Distribution of asymmetry values between pairs of clusters (n = 48) in which only one cluster (marked as c2 in the inset) is connected to additional clusters (marked as x in the inset). c2 is connected to the rest of the network through one link (white bars), or more than one link (black bars). Negative asymmetry corresponds to activity propagation from c2 to c1. Inset: Network schematics. (F) Activation delays as function of time between two specific coupled clusters. Delays were calculated from the location of the cross-correlation function peak, for consecutive NBs during a ten-hour recording (only NBs activating both clusters were analyzed). The black line represents the average delay in one-hour windows. Color coding represents the asymmetry for consecutive NBs.

Fig 4A shows an example of 100 consecutive NBs recorded from a three cluster chain (only NBs in which all three clusters were active are shown). Visual inspection clarified that most of the time cluster 2 fired before cluster 3, indicating that propagation in cluster chains is asymmetric. To examine the different propagation patterns and their abundance in this network, we clustered them by calculating the similarity matrix between NBs using a dendrogram [38] (see Materials and Methods) (Fig 4B). For clarity, only NBs in which at least two clusters were active are presented. Interestingly, clear NB groups with high similarity are observed (marked by solid line rectangles in Fig 4B). These different groups correspond to different propagation patterns as indicated by the average NB profile within each group (Fig 4C). Propagation in this modular network, although bi-directional, is rarely symmetric.

The non-uniformity of the similarity matrix within each rectangle represents NB pattern variation within each group, as seen by examining the coefficient of variation of off-diagonal terms within a group (0.21, 0.04, 0.21, 0.03 and 0.07 in groups 1 to 5 respectively). The higher coefficient of variation in groups involving the activation of cluster 1 suggests that specific links in the network are more variable than others. For example, groups 2A and 2B (see Fig 4C) are characterized by the same propagation directions, but with a different average delay (between clusters 1 and 2). The activation probability of different propagation patterns vary considerably (P = 0.137, 0.673, 0.103, 0.005, 0.082 for groups 1 to 5 respectively). Such variability can be represented by the pattern entropy (*E* = −∑_*i*_*P*_*i*_*log*_2_*P*_*i*_ = 1.006 for this example) as discussed in detail in the next section.

Clustering into propagation groups existed in all network examined (69 chains form 26 cultures). However, this grouping was highly variable between networks both in terms of separability between groups and the number of groups. Consequently, to reliably quantify asymmetry, we needed a measure that reduces the complexity of patterns to simple asymmetric relations between pairs. To do so, we used the cross-correlation function between connected clusters over long recordings of eight hours (Fig 4D). We subtracted the integral over the positive side of the cross-correlation function from its negative side and divided it by the total sum. We analyzed only positive correlations between clusters, thus negative cross-correlation values were not included in the integral. The resulting long-term asymmetry is a measure between -1 and 1 with the sign determining the propagation direction and the value corresponding to the asymmetry magnitude. For example, in Fig 4A–4C, propagation pattern 2 dominated the network's activity (Fig 4B). This pattern represents events initiated in cluster #2 and propagated to the neighboring clusters. Correspondingly, the asymmetry measure for cluster 1 and 2 is -0.21 and for cluster 2 and 3 is 0.31. Pooling over all cluster pairs (n = 89) we find that the average absolute asymmetry was 0.26±0.21 (mean ± standard deviation). Such asymmetry is in accordance with previous reports indicating that activity asymmetry exists between connected sub-populations, even if they are very similar to each other [32]. We next examined if asymmetry is affected by the relative differences in cell number between connected clusters. Although the differences between cell numbers were not large (S1B Fig), we found a small positive correlation between the normalized cell count and the asymmetry value (C = 0.331, PV = 0.001) (S1D Fig).

An interesting question that arises from these results is whether and how activity asymmetry is affected by asymmetry in the cluster pair, and by the manner in which this cluster pair is connected to the rest of the network. To address this question, we applied the asymmetry measure to pairs of clusters in which only one of the clusters was connected to a larger clustered network. Such pairs had a clear structural asymmetry. A schematic drawing of such pairs is shown in the inset of Fig 4E, where c1 and c2 are the two clusters and x denotes a group of connected clusters. We differentiated between two connectivity patterns according to the number of links (connections to other clusters), n, cluster c2 had. The asymmetry statistics for n = 1 and n>1 are shown in white bars and black bars respectively in Fig 4E. In most networks, the long-term activity asymmetry was negative (Fig 4E), corresponding to activity propagation from the cluster group towards the chain's end (x towards c1 in the schematic drawing in Fig 4E). This result suggests that the structural asymmetry of the embedding network contributed to the functional asymmetry of the cluster pair. Thus, if a cluster in a pair is connected to other clusters, it will more likely drive activity in this pair. Interestingly, the average asymmetry of pairs connected through more than one link was more negative than that of pairs connected through one link (-0.26 and -0.10 respectively, PV = 0.07, t-test), suggesting that the drive is stronger when the structural asymmetry is stronger. We note that asymmetry was calculated on long time series to represent a gross averaged estimation in a valid manner. When inspecting the delay of the cross-correlation function (or the asymmetry) on a single cluster pair, we find that asymmetry is modulated in the network (Fig 4F). This is observed in the ratio between propagation to one and to the opposite direction calculated on one hour windows (black line in Fig 4F). Despite this dynamic change, the average values of asymmetry were mostly negative (Fig 4E).

### Control over conditional propagation by disinhibition

**Fig 5:**
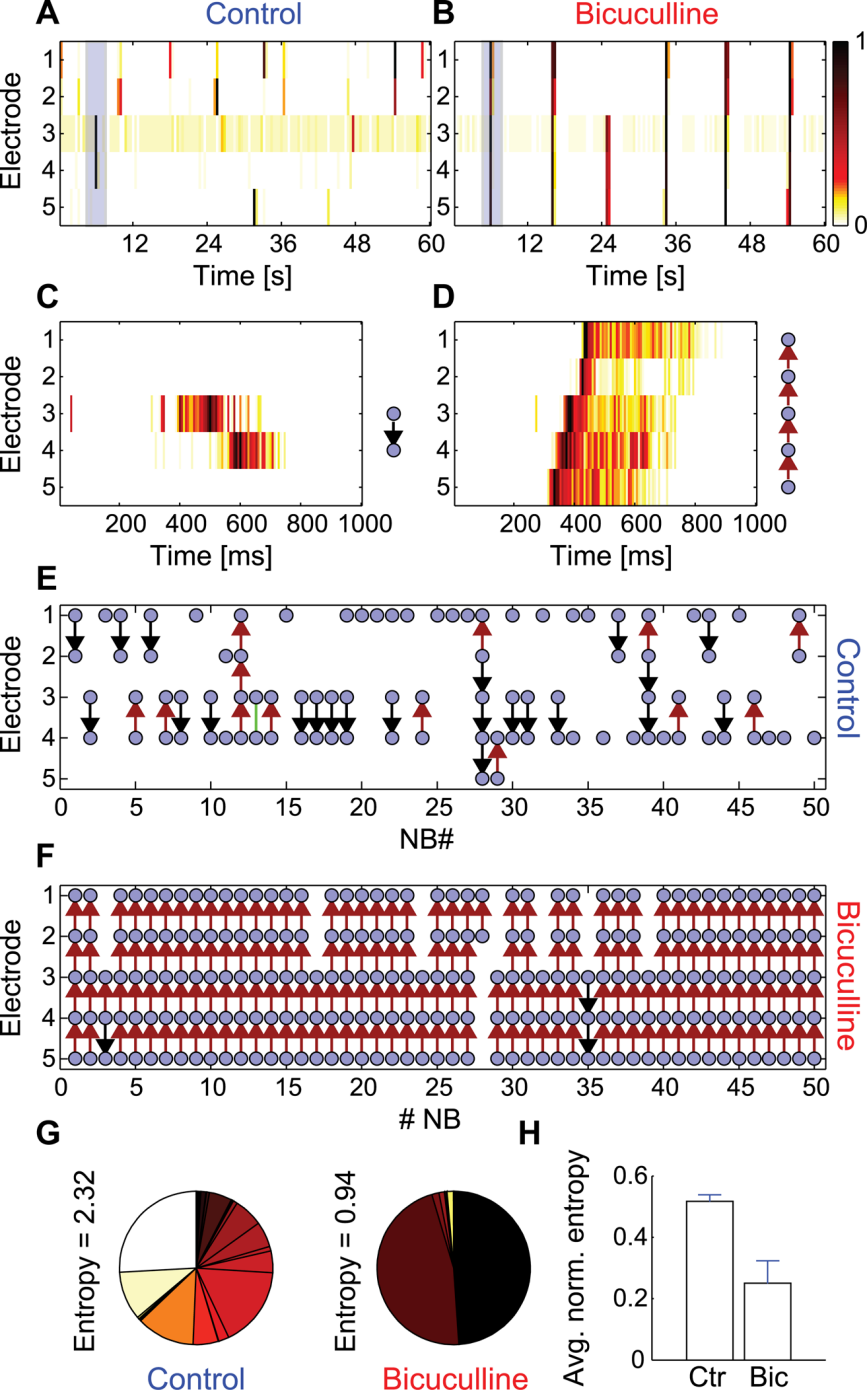
Gating by disinhibition in modular networks. (A, B) Activity intensity raster recorded from a chain of five clusters during control and disinhibition conditions respectively. (C, D) A zoom into (blue rectangles) one of the NBs in (A) and (B) respectively. Each pattern is represented by a schematic drawing, where a blue dot corresponds to the activation of the cluster during the NB, and the arrow corresponds to the propagation direction (red-upwards, black-downwards, green-simultaneous activation). (E, F) A schematic representation of 50 consecutive NB patterns under control and disinhibition conditions respectively. (G) A pie plot of the probability of occurrence of different NB patterns (4097 consecutive NBs) during control (left) and disinhibition (right). The entropy is calculated for each of the pie plots (the same number of consecutive NBs was analyzed under control and Bicuculline conditions). (H) Average normalized entropy in control (Ctr) versus disinhibition (Bic) conditions. Error bars represent the standard errors (n = 6).

We further examined whether we can gate activity propagation by global disinhibition. Specifically, whether we can dramatically increase the probability that NB propagates between clusters by applying inhibitory synaptic blockers (Fig 5). Under control conditions (normal growth media), cluster chains exhibited a large spectrum of activation profiles from confined bursts (in single clusters) to network-wide activation (Fig 5A). Upon disinhibition, using a GABA_A_ (γ-aminobutyric acid) channel antagonist (Bicuculline, 30μM), the conditional propagation was replaced by network-wide synchrony (Fig 5B). Careful examination of the propagation patterns within the NBs under disinhibition revealed that not only did the network synchronize to operate as a single unit (Fig 5B), but also the synchrony was characterized by highly ordered propagation patterns (Fig 5D). To monitor these patterns over consecutive NBs, we represented each NB as a vector (as shown in Fig 5C and 5D-right) and plotted it, for consecutive NBs, under control conditions (Fig 5E) and under disinhibition (Fig 5F). In Fig 5E and 5F, blue dots correspond to cluster activation during an NB, and the arrows correspond to the activity propagation direction (extracted from the peak lag of the cross correlation function). Under disinhibition, the network’s activity collapsed to a stereotypic pattern in which the entire network was fully activated and cluster number 5 functioned as an activation focus. Under disinhibition, 95% of the NBs (N_total_ = 4097) were initiated by cluster 5, in contrast to only 4% (N_total_ = 7206) under control conditions. Such a repeated propagation pattern is in contrast with the much wider repertoire observed under control conditions (Fig 5E).

The variability in NB patterns was further quantified over long-term recordings by defining the NB pattern entropy. Each NB was reduced to a binary series of zeros and ones corresponding to the activation of different clusters (blue dots in Fig 5E and 5F). The occurrence probability for every binary pattern, *P*_*i*_, was calculated and presented as a pie chart for control and disinhibition conditions (Fig 5G). The entropy, *E*, was calculated from these probabilities as *E* = −∑_*i*_*P*_*i*_*log*_2_*P*_*i*_. Under control conditions, a wide NB pattern distribution and relatively high entropy value (*E* = 2.32) were observed. However, after disinhibition, the number of patterns dramatically decreased, corresponding to a lower entropy (*E* = 0.94). For entropy calculations, we used only long enough chains (three or more active clusters) that can support a high enough variability in NB patterns. Since every chain was of a different length and consequently had different potential entropy values, the entropy was normalized to the maximal possible entropy in the chain, *E*_*max*_ = *log*_2_(2^*N*^ − 1) (the term -1 was added to subtract the case in which no cluster was activated). Five out of six chains exhibited a decrease in normalized entropy following disinhibition (Fig 5H). This transition from a wide to a narrow pattern distribution following disinhibition suggests that under control conditions, networks maintain a certain relation between inhibition and excitation. This relation, in combination with the modular architecture, allowed each cluster to be activated autonomously while still being connected to other clusters, and thus also having the potential to activate them. When disinhibited, the network lost its diversity and collapsed into stereotypic global activation. Disinhibition (implemented here using Bicuculline) served as a gating mechanism, altering signal propagation.

### Functional properties of modularity are maintained in large networks of connected clusters

**Fig 6:**
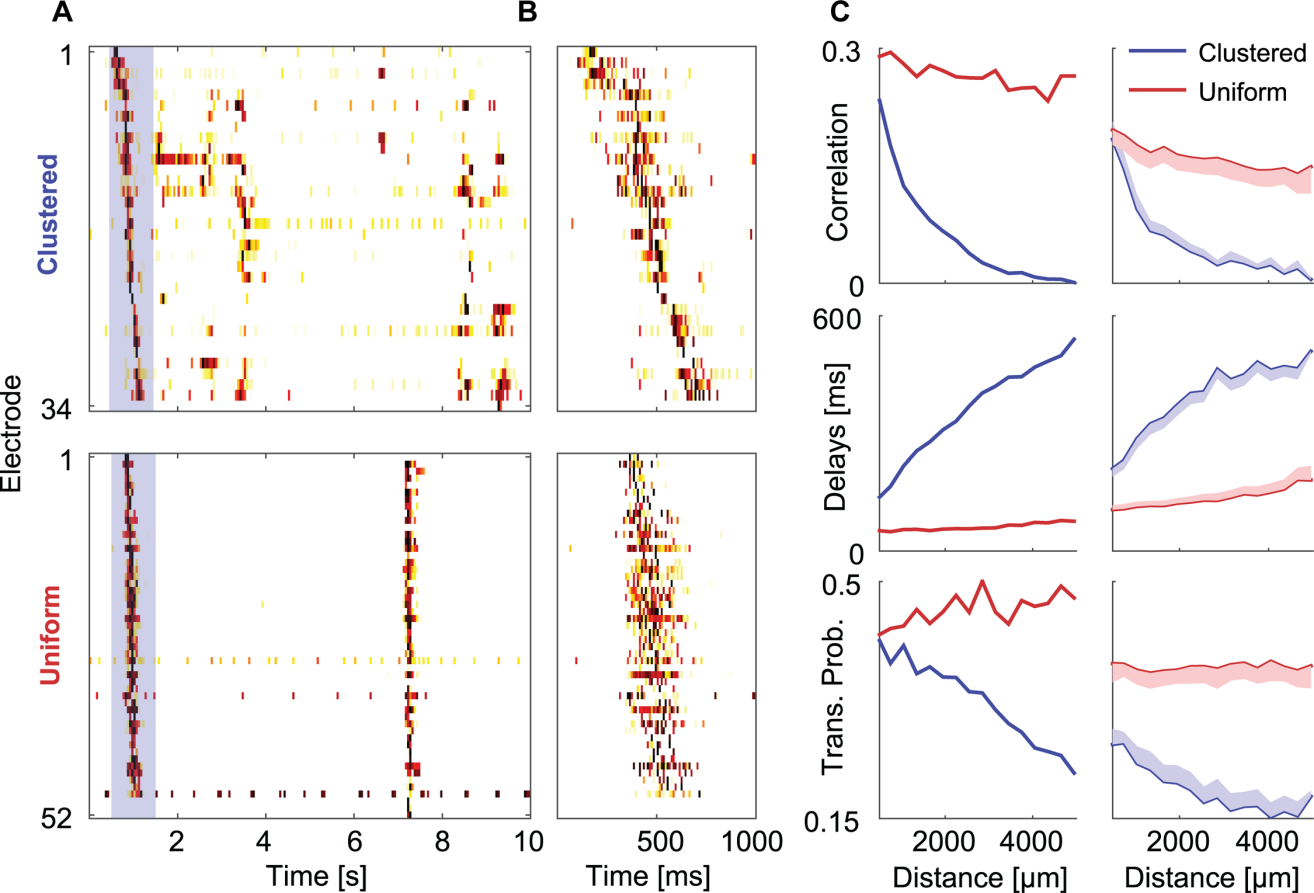
Activity in large modular networks. (A) Activity intensity raster plots recorded from a clustered network (top) and a uniform network (bottom). Electrodes were reordered according to the order of activation (center of mass of the activity in each electrode) during the first network event (blue rectangle). (B) Zoom into the first network event (blue rectangle) from the modular (top) and uniform (bottom) networks. (C) Average correlations (top), activation delays (middle) and NB transfer probabilities (bottom) recorded from different electrodes as a function of the distance between them. Blue and red lines correspond to modular and uniform networks respectively. Left column shows the average over all electrode pairs from the two networks in (A). Distances were binned and the average profile was calculated for all electrode pairs within the bin. Right column is an average over the average profiles of all networks (15 clustered networks and eight uniform networks). Shaded areas correspond to standard errors.

As illustrated above, modularity introduces new features to the activity repertoire of uniform networks. To explore whether these features are preserved in networks of many connected clusters, we examined large, two-dimensional networks of connected clusters (Fig 1C) and contrasted their activity with that of large uniform networks (Fig 1A). The most conspicuous difference appeared to be the degree of synchrony. While uniform networks mostly showed network-wide activation that spanned a large fraction of the cell population (Fig 6A-bottom; S5 Fig), clustered networks exhibited NBs of different sizes that propagated to different distances (Fig 6A-top; S5 Fig). This diversity is the direct result of the conditional propagation inherent in the modular bridge between clusters. In large modular networks, the cumulative number of such bridges between any two clusters increased with the distance between them. We next examined whether co-activation of clusters depended on distance. This effect was quantified by measuring the average Pearson correlation between the activity of cluster pairs for long time periods (>8 hours). A decrease in correlation with distance was evident (Fig 6C-top), highlighting the locality of the activation in large modular networks. Neighboring clusters had a higher probability to fire in synchrony. The tendency for local activation was found to coexist with epochs of global activation (Fig 6A and 6B-top) exemplifying the network's potential for activation diversity.

Large clustered networks were also characterized by long delays. Delays between cluster pairs were calculated from the peak of the smoothed cross-correlation function and averaged over all NBs (see Fig 3). These delays accumulated during burst propagation, and in many cases the last cluster to be activated during an NB began its firing long after the first cluster already ceased bursting (Fig 6B-top). Delays ranged from tens to hundreds of milliseconds (corresponding to an average propagation speed of 6.5±0.2 μm/ms, mean±SE), and increased with the number of bridges between clusters (Fig 6C-middle). Time delays partially affected the decrease in correlation with distance (Fig 6C-top). Conditional propagation was also quantified by calculating the transfer probability. Namely, given the occurrence of an NB in one cluster, the probability that an NB occurred within one second in the other cluster. This probability was also found to decrease with distance (Fig 6C-bottom).

Finally, we note that conditional propagation, long delays and high diversity in the network degree of activation and synchrony are much less pronounced in the activity repertoire of large uniform networks. Uniform networks are characterized by large scale network events (S5 Fig). Once a network event is initiated, it quickly propagates (with an average propagation speed of 22.1±1.2 μm/ms, mean±SE) and recruits most of the network (Fig 6A, 6B-bottom; S5 Fig). Uniform topology appears to lead to uniform activation which does not vary much with distance (Fig 6C). Previous studies showed that uniform networks support a mode of partial network activation called aborted bursts, during which only a subset of the population is active [39]. Owing to these aborted bursts (which are also observed in our data) the average transfer probability in uniform networks is well below unity (Fig 6C-bottom). However, in uniform networks such aborted bursts are not confined to a local area in the network and are always activated in the same sub-population of neurons [39]. Consequently, neither the transfer probability nor the delays in uniform networks depend dramatically on distance (Fig 6C-middle).

### Disinhibition in large networks collapses activity to network-wide stereotypic patterns

**Fig 7:**
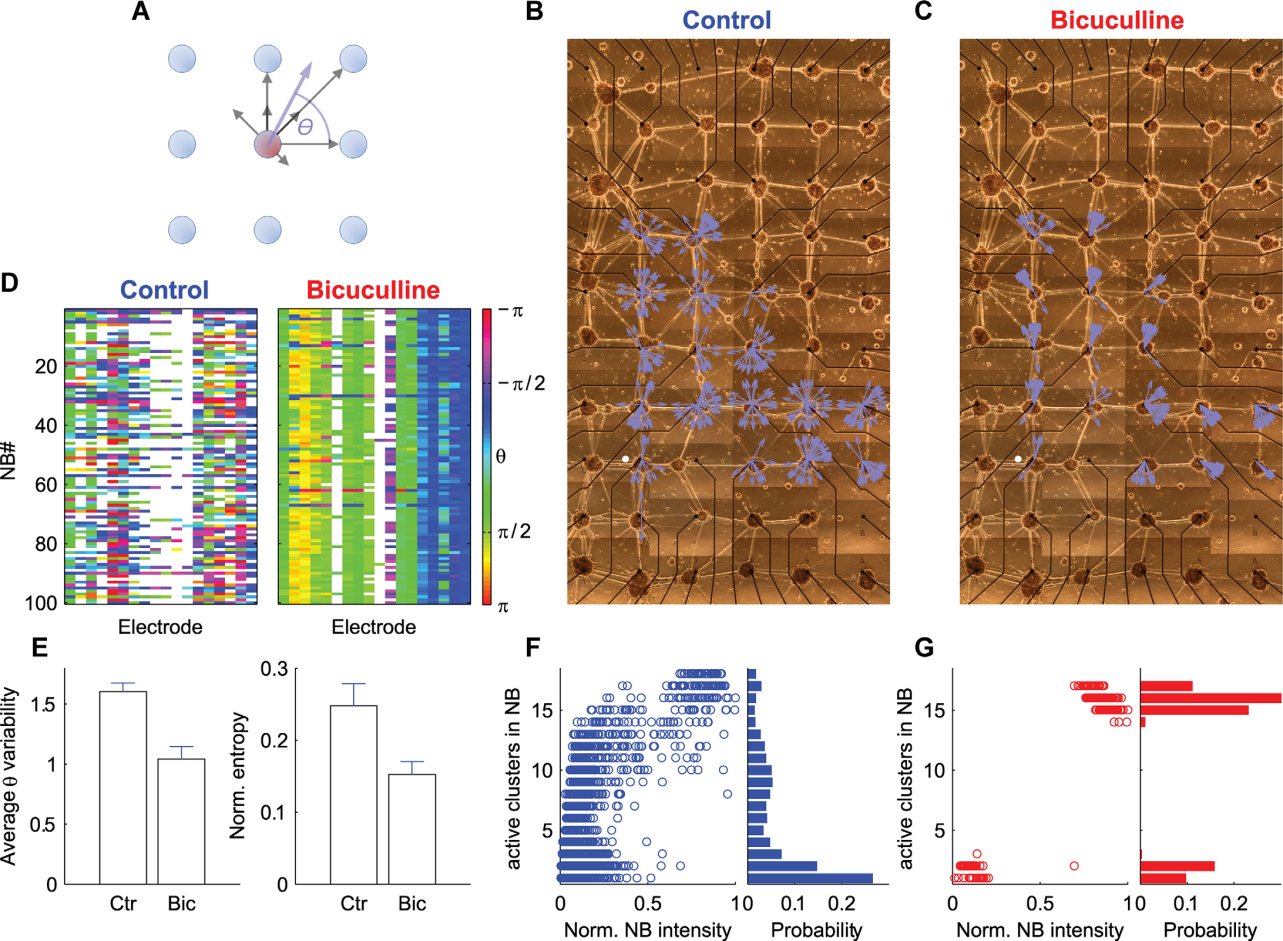
Disinhibition reduces activation diversity in large modular network. (A) Schematic illustration of the propagation vector. The activity flow between a cluster (red) and each of its nearest neighbors (blue) is represented by a vector (gray arrow). The vector direction corresponds to the direction between clusters and magnitude corresponds to the location of the peak of the cross-correlation function (see text for details). Averaging these vectors over all clusters yields a propagation vector for this cluster during a specific NB (blue arrow). The angle of this vector with the positive x-axis is marked by ϴ. (B, C) The propagation vectors (blue arrows) for 20 consecutive NBs plotted on the physical space of the clustered network they were recorded from, before and after application of Bicuculline respectively. The direction of activity flow is represented by the angle, ϴ, of the vector (the magnitude of the vector was set to be equal for all clusters). (D) The propagation vector angle, ϴ, for the clusters in (B, C) during 100 consecutive NBs under control (left) and Bicuculline conditions (right). The decreased variability in ϴ under Bicuculline corresponds to the decreased number of propagation patterns in the network. (E) Statistics for NB activation and propagation variability before (Ctr, Control) and after application of Bicuculline (Bic) (n = 6 networks). Left–average propagation variability over networks quantified by averaging the standard deviation of ϴ over all clusters in each network. Right–average activation pattern variability over networks quantified by the normalized activation entropy (see Fig 5), (the same number of consecutive NBs was analyzed in control and Bicuculline conditions). Error bars in (E) correspond to standard errors. (F) Left–the number of active clusters during different NBs as a function of the normalized NB intensity (NB intensity divided by the number of active clusters) under control conditions. Normalized NB intensity was further divided by its maximal value over all NBs to delimit values between zero and one. Right–distribution of the number of active clusters over different NBs. (G) The same as (F) but for Bicuculline conditions. Only NBs with at least five active clusters were considered (except in (F) and (G)).

As shown above, disinhibition drastically increased propagation between the modular units and induced global network synchronization with defined propagation patterns. In large clustered networks, clusters are connected through multiple pathways which may lead to different disinhibition effects. To test the propagation patterns in large clustered networks before and after disinhibition, we examined the cross-correlations function between clusters over single NBs. Since these are two-dimensional networks (unlike the one-dimensional cluster chains discussed above), we extended our propagation analysis by calculating the propagation vector for every cluster. We first identified NB windows in the network (see Materials and Methods), and calculated the cross-correlations of smoothed (convolution with a Gaussian, σ = 50ms) activity traces between every cluster and all of its neighbors, corresponding to the fact that long range connections between the clusters were rare due to the grid-like organization of the clusters. Clusters with very weak activity during the NBs (active for less than 10 ms) were not analyzed and only NBs with at least five active clusters were considered. The cross-correlation between every cluster pair was represented by a vector with a magnitude corresponding to the location of the peak of the cross-correlation function, and a direction determined by the physical direction between the clusters (Fig 7A—gray arrows). The propagation vector was calculated by averaging the cross-correlation vectors over all active clusters during each NB (Fig 7A—blue arrow). The angle of this vector with the positive x-axis was marked by ϴ (Fig 7A). The propagation vector for each cluster during 20 consecutive NBs is plotted in the physical space of the network in Fig 7B (magnitude was set to be the same for all clusters). In agreement with the high diversity of activation patterns in clustered networks, different NBs propagated to different directions. This was also evident by examining a larger pool of NBs (Fig 7D-left). Here the angle of the propagation vector, ϴ, was presented in color code for every cluster during consecutive NBs.

As in the case of the cluster chains, following disinhibition (application of 30 μM Bicuculline), the high NB pattern diversity was replaced by stereotypic patterns. This shift (or gating) in activation profile is represented by the narrow distribution of propagation directions for different clusters over consecutive NBs (Fig 7C and 7D-right). Furthermore, the propagation patterns revealed the emergence of a clear activity initiation focus, similar to the case of the cluster chains discussed above (Fig 5E and 5F). Under disinhibition, 42% of NBs (N_total_ = 366) were initiated in the lower left cluster (marked by a white dot in Fig 7C), in contrast to only 0.4% (N_total_ = 2345) under control conditions. The propagation variability of the network was quantified by calculating the standard deviation of ϴ for all NBs in the recording, followed by averaging over all clusters. Under control conditions the propagation variability was 1.367 radians, and after disinhibition it decreased to 0.775 radians. The mean propagation variability for different networks is presented in Fig 7E-left. In all analyzed networks (six out of six) a similar reduction in angle distribution was observed. Reduced variability was also observed in the number of participating clusters during NBs. While in control conditions, the NB sizes varied considerably from activation of single clusters to activation of the whole network (Fig 7F-right); after disinhibition, most of the NBs were synchronized over the entire network (Fig 7G-right). The variability in cluster activation was quantified by calculating the entropy of cluster activation patterns, as previously described for cluster chains. All analyzed networks (six out of six) showed a decrease in activation entropy following disinhibition (7E-right).

In Fig 7F and 7G, we explored the number of participating clusters in NBs with different intensities. We plotted the number of active clusters as a function of NB intensity for control (Fig 7F-left) and disinhibition (Fig 7G-left) conditions. In these plots, the intensity of each NB was normalized to the number of active clusters during this NB. Both in control and disinhibition conditions, the total normalized network intensity increased with the number of participating clusters. Thus, the extent of global activation (the number of clusters recruited during the NB) affected the local degree of activation (NB intensity in single clusters). However, under disinhibition most NBs recruited the entire network, while in control conditions, different NBs recruited a different number of clusters (Fig 7F and 7G-left). This is further illustrated by plotting the distribution of the number of recruited clusters over all NBs in control (Fig 7F-right) and disinhibition (Fig 7G-right) conditions.

### Gating by disinhibition requires modular topology

The results described above demonstrate that modular topology support gating by disinhibition. To better understand this effect, we developed a computational model based on two coupled clusters in which different network parameters could be systematically modified. As cortical cultured networks exhibit complex organization of dynamical patterns, we adopted a previously published model that reproduced the main features of these patterns [32,40]. Neurons (N = 50 in each cluster) were modeled as Morris-Lecar elements with modified Tsodyks-Markram synapses and synaptic noise (see Materials and Methods). One out of every five neurons is an inhibitory neuron. For isolated clusters, the connectivity probability within clusters (intra-connectivity) was 0.25 and 0.2 for clusters 1 and 2 respectively, and the connectivity between clusters (inter-cluster connectivity) was initially set to zero. Intra-cluster connections were then replaced with inter-cluster connections with a probability λ which is defined as the modularity of the system. At the limit of λ = 0.5 the two clusters converge to a large uniform network. The model parameters (see Materials and Methods) were chosen to fit experimental data.

**Fig 8:**
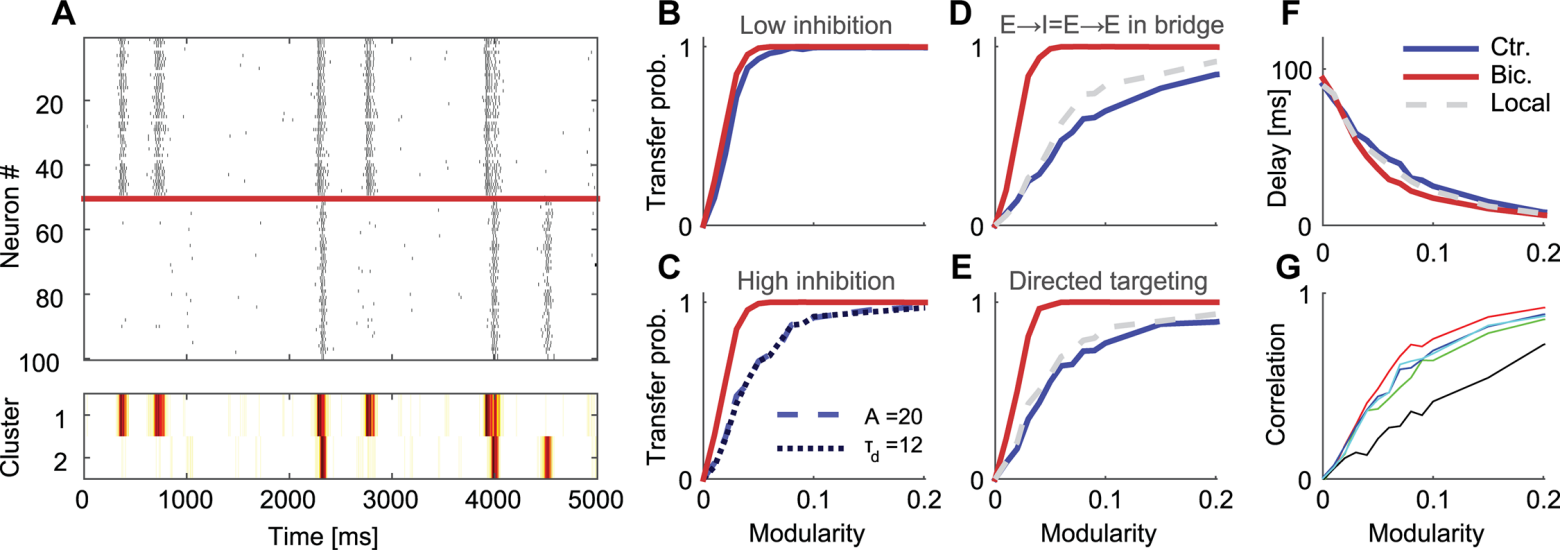
Model of activity gating in modular networks. (A) Top–A raster plot from a simulation of two coupled clusters (λ = 0.02). Bottom—Activity intensity of the raster plot in (A). (B-E) The transfer probability (the average probability that an NB initiated in cluster 1 will propagate to cluster 2 as a function of modularity). The blue, red and dotted gray curves correspond to control, Bicuculline (inhibitory synapses blocked) and local inhibition (inhibitory neurons from one cluster do not connect to the other cluster) conditions respectively. (B) Low synaptic strength for inhibitory neurons (A = 10). (C) High synaptic strength for inhibitory neurons (τ_d_ = 6, A = 20, light blue), and high synaptic depression time constant for inhibitory neurons (τ_d_ = 12, A = 10, dark blue). (D) Strong feed-forward inhibition—excitatory neurons contact the same number of excitatory and inhibitory neurons in the other cluster (τ_d_ = 6, A = 20). (E) Direct targeting inhibition—inhibitory neurons that receive excitatory input from the other cluster preferentially target excitatory neurons in the same cluster (τ_d_ = 6, A = 20). (F) The delay between the activation of cluster 1 and cluster 2 as a function of the inter-cluster connectivity. (G) Correlation between the total firing rate in the sending and receiving clusters over different bursts (red, green, blue, black and cyan curves correspond to control conditions in B, light blue curve in C, dark blue curve in C, dark blue curve in D, and dark blue curve in E respectively). The results of B-G were averaged over 20 simulations each with a different network realization.

When the inter-connectivity was set to zero (isolated clusters), expectedly, each cluster exhibited short epochs of network bursts that were separated by sporadic single neuron activation, similar to activity patterns of isolated clusters in culture [29]. For slightly larger inter-cluster connectivity (λ = 0.02), some NBs successfully recruited the connected cluster, while others failed to elicit an NB in the connected cluster (Fig 8A). To quantify this property, we calculated the transfer probability for different modularity values. To do so, we detected NBs in the two clusters. An NB was considered as transmitted if following the activation of cluster 1, an NB peak was detected in cluster 2 within a time frame of 200 ms (see Materials and Methods). Since both clusters were spontaneously active, the transfer probability was non-zero even if the clusters were disconnected (λ = 0). To compensate for this, the number of “transferred” NBs at λ = 0 was subtracted from the measured number of transferred NBs and the total number of fired NBs before calculating the transfer probability.

We further examined how the transfer probability depends on the strength of inhibitory synapses [41]. For low inhibition levels, the transfer probability (Fig 8B) was highly dependent on the inter-cluster connectivity and changed between 0 and 1 (full transmission), indicating that modularity directly controls transmission probability. However, this modulation occurred over a narrow λ range and was insensitive to disinhibition (eliminating inhibitory synapses analogous to globally applying Bicuculline to the in vitro network) (Fig 8B). Increasing the decay time constant for inhibitory synapses (τ_d_), or the inhibitory synaptic strength (A) twofold, increased both the transition range and the sensitivity to inhibition block (Fig 8C). This suggests that various mechanisms that increase inhibition within the time scale of an NB, such as selective increase in synaptic strength, or neuromodulation of synaptic decay dynamics, can support gating by disinhibition.

We next tried to identify other putative mechanisms which can increase disinhibitory effects in modular networks by focusing on the properties of the bridge connecting the two clusters [4]. We set the inter-cluster connection probability between pre-synaptic excitatory and post-synaptic inhibitory neurons to be as high as the excitatory to excitatory probability. This dramatically increased the range over which transmission was modulated (Fig 8D). Furthermore, following disinhibition, the transfer probability dramatically increased (Fig 8D—red curve), opening the gate between the two clusters. Thus, under these settings, our model captured the conditional propagation between sub-populations in a modular network and the gating of this conditional propagation by disinhibition.

An alternative to increasing inhibition strength is targeting inhibition, for example, by selectively directing inhibition to excitatory neurons. The existence of such targeting was verified both in the cortex and other brain regions [42,43]. Specifically, we examined whether it is possible to control transmission by selectively directing the output of inhibitory neurons, which receive inter-cluster input, to excitatory neurons (see detailed connectivity schemes in Materials and Methods). Indeed, doing so increased the control over transmission to almost the same extent as when increasing the feed-forward inhibition (Fig 8E). Interestingly, the two mechanisms described above were largely insensitive to the elimination of inhibitory connection through the bridge (Fig 8D and 8E–gray line), congruous with the notion of local inhibition.

Similar to the in vitro modular networks described above, the propagation of activity between connected sub-populations in the model was characterized by long delays (calculated as the time lag between the locations of NB peaks in both clusters, see Materials and Methods). Delays of several tens of milliseconds decreased with the connectivity between clusters (Fig 8F—blue curve). These delays were independent of the strength of inhibitory drive or whether it was removed altogether, as in the case under inhibitory block, suggesting that they are a consequence of the modular organization (see Discussion).

Finally, we examined whether information about the firing rate is transmitted through the bridge between the two sub-populations, as in our in vitro experiments. We analyzed the correlations between the total firing rates in the sending cluster as a function of the firing rate in the receiving cluster (similar to Fig 3D). We found that the correlation increases as a function of modularity (Fig 8G) and begins to saturate for λ = 0.1.

## Discussion

In this study, we systematically examined, experimentally and theoretically, the effect of network modularity on activity transmission between neuronal assemblies. A clear hallmark of the modular networks we studied is their capacity to support long delays in the order of 100 milliseconds (corresponding to a 5 μm/ms velocity between connected clusters) (Figs 3E and 6C-middle). Since axonal propagation speeds in culture are fast (>200 μm/ms, [44]), the observed delays are likely to be associated with the time it takes the receiving cluster to generate a network response due to multiple synaptic delays (recruitment time) (S3 Fig; for a more elaborate discussion see [39]). These delays are shorter on average in large networks of connected clusters, presumably due to the increased number of pathways between any two clusters, but are still much longer than in uniform networks.

Why is the capacity to support long delays useful? Foremost, long delays give rise to time scale separation between the activities of different modules and are a means to dissociate the intra-module from the inter-module processing [21]. Interestingly, our networks (Fig 3E-right) showed high variability in the average delays between different networks. This variability may stem from network architecture variability, and implies that modularity has the potential to support variable delays. Indeed, our model shows that delays can be controlled by modifying the coupling between sub-populations (Fig 8F). In a previous report (performed under the same experimental conditions as here) we showed that increasing the coupling in modular networks resulted in shorter delays [45]. We also observed variability in delays within specific networks over time (Fig 3E-left), implying that delays may be dynamically regulated to control transmission, for example by short- term plasticity [46].

An additional property of modular circuits is their activation asymmetry. Asymmetry may be important for controlling transmission directionality. In cluster chains, asymmetry was manifested by propagation patterns which were more probable than others (Fig 4A–4C). It was previously reported that coupled networks of similar sizes exhibit inherent asymmetry, and that this asymmetry is associated with the structural asymmetry of the connecting bridge [32]. Accordingly, a small subset of neurons at the bridge controls the propagation between networks. Our results support these previous findings, but suggest that functional asymmetry is also affected by the manner by which the coupled network is embedded within a larger network. When a network of coupled clusters was connected to a larger network, activity mostly propagated from the cluster connecting the large network to its neighbor (Fig 4E). Furthermore, higher asymmetry in the embedding network resulted in higher asymmetry between coupled clusters (Fig 4E). Interestingly, inhibition played a major role in determining asymmetry. Blocking the inhibition drastically affected the propagation direction between connected clusters (Fig 5E vs. 5F), suggesting that transmission directionality can be modulated to a large extent by reducing inhibition. However, further experiments, in which the cluster composition (e.g. the number of excitatory vs. inhibitory neurons) is monitored, are required to understand the morphological basis of asymmetry and transmission.

Neural networks have to maintain a fine balance between segregated activation (where activity is restricted to a specific sub-population) and integrated activation (where activity spreads to connected sub-populations) [9,47]. In the brain, functional segregation is associated with the structural modularity of the circuit [10], and the balance between functional segregation and integration explains the high functional complexity in the network [19,22,48,49]. We have shown that a similar fundamental property exists in small modular circuits. Namely, networks support a wide variety of activations from activity epochs which are confined to one sub-population to large-scale activation of the entire network. We observed such features both in one-dimensional cluster chains (Fig 5A and 5E), and in two-dimensional clustered networks (Figs 6A and 6C-top and 7B and 7F), but to a much lesser extent in uniform networks (Fig 6A, C-bottom). We found that both the number and intensity of activated clusters (Figs 5E, 5G, 7E and 7F), as well as the propagation direction (Figs 5B, 5D, 7B and 7E), were highly variable between consecutive NBs. In addition, due to long delays, this spatial activation variability resulted in temporal variability of NB durations. Interestingly, both temporal and spatial variability were previously reported for small-scale circuits in cortical slices. Organotypic slices show avalanche-like patterns typified by a wide distribution (heavy tail) of event sizes and durations [50]. The hypothesis that this diversity reflects the transient activation of different cell assemblies is supported by our results.

We suggest that the activation diversity in our networks is the outcome of the conditional propagation between sparsely coupled clusters. In small modular networks, activation of one sub-population does not necessarily lead to the activation of the other (Fig 3B and 3C). Our model supports this idea and illustrates how conditional propagation can be controlled by changing the degree or architecture of coupling between sub-populations (Fig 8C–8E). We note that we focus here only on a specific dimension of activation diversity. Uniform networks exhibit rich dynamical behavior along many spatial and temporal degrees of freedom [38,39,51,52]. However, a fundamental property of their activity is network synchrony on a time scale of ~100 ms [39]. Each of our clusters exhibits similar activity profiles to uniform networks [29], but the weak connections between clusters allows to spatially and temporally decouple their activity on this time scale.

We further suggest that the fact that conditional propagation spontaneously emerges in modular networks (in contrast to uniform networks) is associated with the networks’ self-regulation. We previously showed that isolated clusters of different sizes, and different connectivity, sustain moderate activity levels [29]. This corresponds to well-documented reports of structural and functional self-regulation of excitability in neurons [41,53–55]. Thus, neurons increase or reduce their propensity to fire when activity levels are low or high respectively. However, since in modular networks the fraction of connections between sub-populations is lower than within sub-populations, moderate activity in a cluster may still be below the self-regulated activation limit in the connected cluster (assuming that all sub-populations employ similar self-regulation mechanisms), resulting in the threshold-like behavior we observed (Fig 3C and 3D). Thus, one possible mechanism for transient increase in transmission is by increasing firing rates in the sending cluster, which may be one of the components contributing to transmission under disinhibition. Conversely, the post-synaptic currents to neurons in the receiving cluster at the time of the burst are another factor determining transmission. In principle, such currents can be modulated by neuromodulators or by a third cluster impinging on the receiving cluster. The latter is expected to be more prominent as the number of connections in the network increase. Indeed, while in one-dimensional chains, activity slightly decayed as it propagated between connected pairs, contributing to propagation failure, in two-dimensional clustered networks, a considerable fraction of events still managed to propagate to a large fraction of the network (S5A Fig). This may suggest that transmission in these two-dimensional networks is enhanced by the fact that each cluster is connected to several clusters, thus increasing the probability that their simultaneous activation recruits the receiving cluster. Further studies of single clusters receiving convergent input from two or more controlled clusters are required to explore activity integration in such networks.

By design, our modular networks were spatially regular (Fig 1C). Such a design minimized the variability in connections between clusters and variability in cluster sizes (S1B Fig), since self-organization is constrained by the regular pattern. This allowed us to focus on effects of modularity while partially avoiding the influences of other topological properties. For example, it was shown that if clusters freely organize without spatial constraints, cultures drive themselves towards assortative topologies with a “rich-club” core [56]. Such an organization equips modular networks with additional functional features, such as higher resilience to network damage compared to uniform networks. We previously reported [45] that modular networks with strong connectivity between modules do not give rise to long delays and conditional propagation. In addition, uniform networks in cultures are rarely uniform [51,52], and the uniformity probably depends on the level of granularity at which connectivity is examined. For this reason, we focus here on the extreme case of high intra-cluster connectivity with weak inter-cluster connectivity. Only at this level do we see a marked transition in the network’s activity profiles.

We showed that disinhibiting networks allows us to control transmission through modular circuits (Figs 5 and 7). Disinhibiting the network replaced the conditional propagation with reliable transmission (Figs 5A–5E, 7F and 7G). Not only was the diversity in the number of activated clusters removed (Figs 5E–5H and 7E–7G), but also the diversity in propagation directions (Figs 5E and 5F, 7D and 7E). These results were reproduced by our model. Blocking inhibitory synapses increased transmission, effectively “opening the gate” between connected modules (Fig 8D and 8E). Interestingly, this effect was weaker when the inter-cluster connectivity scheme was similar to the intra-cluster scheme (Fig 8C), suggesting that either stronger feed-forward (Fig 8D) or targeted (Fig 8E) inhibition may be instrumental in controlling propagation.

In addition to increased transmission, blocking inhibition in modular networks led to the emergence of an activation focus, which was absent before disinhibition (Figs 5F and 7B). The global disinhibition we induced in our examinations is used to illustrate the capacity of disinhibition to gate the system between different propagation states. In vivo, the inhibition-excitation ratio can be controlled locally, for example by neuro-modulators [57]. Further studies in which the degree of inhibition is manipulated in selective clusters (for example using Channelrhodopsin and Halorhodopsin) will determine the degree to which such gating can be controlled. Our global disinhibition was used to establish a proof of principle and is more akin to pathological conditions, as in the case of epilepsy where inhibition control is suspected to fail in large neuronal populations [58]. Indeed, under such conditions, the emergence of a focus (Figs 5F and 7B), and the repeated activation waves (Fig 5B), are clear hallmarks [59]. Interestingly, in addition to inhibition deficiency [60], lack of sparse functional connectivity between brain modules was also associated with epilepsy [61].

We note that in previous theoretical studies, gating was investigated in the context of balanced networks showing asynchronous irregular patterns [3,5]. In contrast, our study targets a different regime of population activity patterns. Primarily, our neurons are not constantly driven as the aforementioned model neurons. In such networks, excitatory-inhibitory balance does not result in persistent irregular patterns, but in synchronized bursting behavior. Nevertheless, excitatory-inhibitory balance does exist in these networks and is vital to the networks’ functionality [41]. Consequently, our model system is relevant for investigating gating during increased activity transients as occurring during synchronized and/or bursting activity. Such transient increase in excitability may have a fundamental role in transferring information between different cell populations [17,62,63].

To conclude, it is widely accepted that structure and function are closely related in neuronal circuits. However, the contribution of circuit topology to circuit function often remains hidden due to the difficulty in isolating small circuits in intact tissue. By engineering modular circuits in vitro, we explored the functional consequences of modularity and demonstrated that modular topology and disinhibition are instrumental in gating activity, directly demonstrating how structure can shape function in small neuronal circuits.

## Materials and Methods

### Cell cultures

The entire neo-cortex of (E18-19) Sprague Dawley rat embryos of either sex were removed, chemically digested and mechanically dissociated by trituration, as detailed in a previous publication [29]. Dissociated cells were suspended in a growth medium and plated onto patterned substrates at a density of 700 cells/mm^2^. To promote the long-term cell survivability, a “feeder” colony of cells was added to the culture chamber [64]. The surrounding feeder culture did not directly contact the patterned culture. The mitotic inhibitor, FuDr (80μM FuDr, Sigma, Cat. No. F0503 and 240μM Uridine, Sigma, Cat. No. U3303) was added after four days in culture. Cultures were maintained at 37°C with 5% CO_2_ and 95% humidity. The growth medium was partially replaced every three to four days. The procedure was done in accordance with the NIH standards for care, and use of laboratory animals and was approved by the Tel Aviv University Animal Care and Use Committee. Overall 69 cluster chains (from 26 cultures), 15 large clustered networks, and eight uniform networks were tested in this study.

### Electrophysiology

Extra-cellular recordings were conducted using a low noise pre-amplifier board (MEA1060-BC amplifier, gain ×1,100 with a band-pass filter of 10 Hz to 3 kHz, by Multi Channel Systems, MCS, Reutlingen, Germany). Signals were sampled at 10 kHz and stored on a personal computer equipped with a 60 channel, 12-bits data acquisition board (MC_Card, MCS GmbH), and an MC_Rack data acquisition software (MCS GmbH). An additional 200 Hz high pass filter (2^nd^ order Butterworth) was applied to the data stream by the software. Recordings were performed 12 to 28 days in vitro.

### Network patterning

The patterning method was adapted from a previous publication with slight modifications [27]. Briefly, PDL (Sigma, Cat. No. p7889) islands on top of commercial MEAs (MCS GmbH) were prepared with a soft lithography process using polydimethylsiloxane (PDMS) stencils. An SU8-2075 (MicroChem Corp) mold with approximately 150 μm thickness was casted onto a patterned silicon wafer. The pattern consisted of a rectangular grid (6 x 10) of circles with diameters ranging between 80 and 200 μm with 500 μm spacing. The PDMS stencil was prepared by spin coating the wafer with PDMS. After detaching the PDMS substrate from the mold, the stencil was placed on commercial MEAs and aligned with the electrode locations. The PDL solution was applied to the PDMS stencil and the PDL was dried on a hot plate at 37°C for half an hour. The PDMS stencil was removed before cell plating. The probability of inter-cluster connections depended on island diameter: Larger islands resulted in networks with a higher degree of connectivity. The network's self-organization lasted up to ten days in culture, after which the patterns became stable.

### Activity representation

To quantify network level activity, we calculated the activity intensity (AI) of each cluster:

$$AI=\{\begin{array}{c} A,\;A\geq 0\\ 0,\;A<0 \end{array}\;,\;A=\frac{\sum_{i=1}^{M}\left|V(i)\right|}{M}-NT$$

where V is the voltage waveform, M is the number of samples in each activity intensity bin, and NT is the activity intensity noise threshold. The noise threshold is added to remove the contribution of noise to the AI value and was calculated as follows: The unbiased kurtosis (measuring Gaussianity) of the voltage trace is calculated in time bins of 20 ms. The kurtosis of a univariate Gaussian distribution is 3. Active bins were characterized by super-Gaussian distributions; therefore bins with kurtosis values higher than 3.1 were rejected. The waveforms of the rest of the bins were used to estimate the average absolute value of the noise voltage, which is the noise activity intensity threshold, NT. Once NT is obtained, AI is calculated according to the equation for AI (above). We have previously shown that the activity intensity measure can serve as a good estimate for changes in firing rate of superimposed spikes [29].

### NB detection and parameter extraction

To detect NBs in single channels we used a previously described method [29]. Briefly, we first calculated AI in bins of 2 ms. Next, we counted the number of active AI bins (having non-zero values) in moving windows of length W = 100 ms (steps of 10 ms). W = 100 was chosen because it is long enough to achieve a smooth NBs profile (but not longer than a typical NB). In the model W = 10 was long enough, since the large number of sampled neurons resulted in a smoother profile to begin with. Single sporadic spikes, as well as short threshold crossings, may contaminate NB profiles. As the rate of these events is well below 10Hz, we eliminated them by zeroing data points below a threshold value of T (T = 10). For the model, this value was chosen as T = 5 since the activity is not contaminated by noise. To ensure that the activity near NB edges was included, a second convolution with the same kernel, followed by thresholding with a value of 1, was performed. In the resulting time series, NBs are represented by a series of consecutive positive values. Finally, to ensure that short transient decreases did not result in a separation of NBs to two events, NBs occurring less than G milliseconds apart (end of previous to beginning of next) were merged into one NB (G was set to 100 ms for single clusters in the experimental data and 50 ms for the model in which response variability was lower). To increase the accuracy of the NB start time, end time and peak time detection, the AI function was extracted during the time windows of the previously calculated NB occurrences. The beginning and end of NBs were taken as the first non-zero value from left and right respectively. The peak of the NB is determined by smoothing the AI (using a convolution with a Gaussian, σ = 50 ms), and extracting the time of the maxima. For identifying NBs globally in the whole network, instead of in single channels, events were counted in all channels instead of only in one, and G was set to 1 s (in accordance with the large delays in clustered networks). In addition, channels with very weak activity during each NB (active for less than 10 ms) were not considered. To extract NBs in the computational model, the spike timings of all neurons were counted instead of activity events. The results of NB detection and parameter extraction were verified by manual inspection for all clusters.

### NB similarity matrix

Detection of correlations between bursts was performed using a hierarchical clustering algorithm [38]. Briefly, AI traces were extracted during a 1000 ms window surrounding the detected NB peaks and smoothed by convoluting with a Gaussian (σ = 10 ms). The time invariant correlation between the i^th^ and j^th^ NBs were calculated as follows: $R_{ij}={max}_{t}\{{\left\langle C_{ij}^{n}(t)\right\rangle }_{n}\}$, where $C_{ij}^{n}$ is the normalized cross-covariance between the AI trace during the i^th^ and j^th^ NBs of the n^th^ cluster, and *t* is the time index of the cross-covariance function. To identify groups of similar bursts, *R*_*ij*_ is reordered using the dendrogram hierarchical clustering algorithm. The dendrogram is calculated on the Euclidian distance matrix, *D*_*ij*_, between the i^th^ and j^th^ rows in *R*: *D*_*ij*_^2^ = ∑_*k*_(*R*_*ik*_ − *R*_*jk*_)^2^.

### Computational model

Simulated networks consisted of two clusters, each with 50 Morris-Lecar neurons (see neuron model for details). Neurons were connected through modified Tsodyks-Markram synapses (see synapse model for details). Connectivity was defined by the matrix *a*_*ij*_, where *a*_*ij*_ = 1/0 corresponds to an existing/non-existing connection between the pre-synaptic terminal of neuron i and the post-synaptic terminal of neuron j. One of every five neurons was randomly selected as inhibitory. Each network was simulated for 300 s using an Euler integrator with a 0.1 ms time step.

The modularity of the network was determined by the parameter λ as follows. The connectivity probability within cluster 1 (neurons 1 to 50) and cluster 2 (neurons 51 to 100) was initially set to 0.25 and 0.2 respectively, and the connectivity between clusters was set to 0. Next, we randomly replaced intra-cluster connections with inter-cluster connections with probability λ. Thus, for λ = 0 the network is composed of two isolated (disconnected) clusters, and for λ = 0.5 the intra-cluster connectivity is equal to the inter-cluster connectivity, and the initial separation to two clusters can no longer be observed.

We simulated three connectivity schemes, which differ by the number and distribution of inhibitory (I) and excitatory (E) synapses: proportional inhibition, strong feed-forward inhibition, and direct targeting inhibition. We kept the E/I neuron ratio constant, although in the experimental conditions some variability may occur. Such a choice is consistent with the self-regulation of synaptic transmission which compensates changes in the network structure to maintain E/I balance [41,65].

In general, the connectivity between two neurons can be one of the following: E→E, E→I, I→E and I→I. These schemes were differentiated by ⟨*N*_*v*→*w*,*x*→*y*_⟩ which denotes the expected value of the number of synapses from cluster *v* to *w*, where *x* and *y* stand for the type of the pre and post-synaptic neuron type (E or I). For example, *N*_2→1,*E*→*I*_ is the number of *E* → *I* synapses from cluster 2 to cluster 1.

1. *Proportional inhibition*—the distribution of inter-cluster connections is the same as for the intra-cluster connections. Namely, $\left\langle N_{v\to w,x\to y}\rangle =\frac{\lambda }{1-\lambda }\langle N_{w\to w,x\to y}\right\rangle$ for any combination of *v* ≠ *w*,*x*,*y*.
2. *Strong feed-forward inhibition*–the number of *E* → *I* synapses between clusters is set to the number of *E* → *E* synapses. Namely, ⟨*N*_*v*→*w*,*E*→*I*_⟩:= ⟨*N*_*v*→*w*,*E*→*E*_⟩, for any combination of *v* ≠ *w*. The rest of the synapses are calculated as in the proportional inhibition case.
3. *Direct targeting inhibition*–inhibitory neurons receiving inter-cluster inputs target only excitatory neurons. Namely, for every inhibitory neuron having at least one excitatory input from the other cluster, we replace all synapses to other inhibitory neurons in the same cluster with synapses to randomly selected excitatory neurons from the same cluster.

For the above connectivity schemes, two conditions were used: inhibition block and local inhibition. For inhibition block (analogues to application of Bicuculline), all inhibitory synapses were disabled by setting *A* = 0 (see synapse model). For local inhibition, only inhibitory inter-cluster connections were disabled (*A* = 0).

### Neuron model

Neurons were modeled as Morris Lecar elements [66]:

$$C_{m}\dot{V}=I_{ext}-g_{Ca}M_{SS}(V-V_{Ca})-g_{K}W(V-V_{K})-g_{L}(V-V_{L})$$

$$\dot{W}=\phi (W_{SS}-W)\cosh(\frac{V-V_{3}}{2V_{4}})$$

$$M_{SS}(V)=0.5(1+\tanh\frac{V-V_{1}}{V_{2}})$$

$$W_{SS}(V)=0.5(1+\tanh\frac{V-V_{3}}{V_{4}})$$

where *V* is the membrane potential, *I*_*ext*_ is the externally applied current, *W* and *M* are the fraction of open K^+^ and Ca^+2^ channels respectively, and *C*_*m*_,*ϕ*,*V*_1_,*V*_2_,*V*_3_,*V*_4_,*g*_*k*_,*g*_*Ca*_,*g*_*L*_ are constants, adopted with slight modifications from Rinzel and Ermentrout [66] (see Table 1 for a full list). These parameters were selected to simulate a class I neuron which can generate cellular-level [66] and network-level [40], bursting in accordance with the activity of isolated clusters [29]. We also examined networks of leaky integrate and fire neurons. Although we could qualitatively reproduce our results with these neurons, Morris-Lecar neurons gave a much better fit to the experimental observations (for discussion see [40]). Neurons received both synaptic and noise input: *I*_*ext*_ = *I*_*n*_ + *I*_*syn*_

**Table 1:** Neuron model parameters.

Parameter	Excitatory Neuron	Inhibitory Neuron
*g*_*Ca*_ $\left[\frac{mS}{{cm}^{2}}\right]$	1.0	1.0
*g*_*K*_ $\left[\frac{mS}{{cm}^{2}}\right]$	2	2
*g*_*L*_ $\left[\frac{mS}{{cm}^{2}}\right]$	0.5	0.5
*V*_*Ca*_[mV]	100	100
*V*_*K*_[mV]	−70	−70
*V*_*L*_[mV]	−50	−50
*V*_1_[mV]	−1	−1
*V*_2_[mV]	15	15
*V*_3_[mV]	10	10
*V*_4_[mV]	14.5	14.5
*C*_*m*_ $\left[\frac{\mu F}{{cm}^{2}}\right]$	1	2
*ϕ*	1/3	1/3

### Noise model

The noise, *I*_*n*_, fed into every neuron, was selected from a Gaussian distribution (mean, $\mu =7.55\left[\frac{\mu A}{{cm}^{2}}\right]$ and standard deviation, $\sigma =4[\frac{\mu A}{{cm}^{2}}]$) (independently identically distributed for each neuron) for every simulation step. This choice was driven by the assumption that noise originated from spontaneous synaptic release of neurotransmitter [67]. Such noise could be modeled as an Ornstein–Uhlenbeck process [68]. Considering that for single synapses, the time between spontaneous releases is a Poisson process [69] and that the number of synapses onto a neuron is large, the overall spontaneously evoked noise current can be approximated by a single Gaussian variable (in accordance with the central limit theorem).

### Synapse model

Synapses were modeled as modified Tsodyks-Markram elements [70]:

$$\dot{x}=\frac{z(-\tan(1.2z-1.2))}{\tau_{rec}}-ux\delta (t-t_{AP})$$

$$\dot{y}=-\frac{y}{\tau_{d}}+ux\delta (t-t_{AP})$$

$$\dot{z}=\frac{y}{\tau_{d}}-\frac{z(-\tan(1.2z-1.2))}{\tau_{rec}}$$

where *x*, *y* and *z* are the fractions of synaptic resources in the recovered, active, and inactive states of the synapse. *τ*_*rec*_ and *τ*_*d*_ are time constants representing the recovery and decay of active resources respectively (see Table 2 for a full list). *t*_*AP*_ is the arrival time of the last action potential to the pre-synaptic terminal. *u* is the fraction of resources activated upon action potential arrival. In excitatory synapses, *u* was constant (*u* = *U*_0_). In inhibitory synapses, *u* was a dynamic variable enabling synaptic facilitation during bursts: $\dot{u}=-\frac{u}{\tau_{facil}}+U_{0}(1-u)\delta (t-t_{AP})$

**Table 2:** Synapse model parameters.

Parameter	E->E Synapse	E->I Synapse	I->E Synapse	I->I Synapse
*A*[mV]	6.25	10	-10/20	-10/20
*u* [scalar]	0.2	0.2	Dynamic	Dynamic
*U*_0_ [scalar]	-	-	0.06	0.06
*τ*_*rec*_[ms]	100	100	800	800
*τ*_*d*_ [ms]	6	6	6/12	6/12
*τ*_*facil*_ [ms]	-	-	1000	1000

We modified the *x* and *z* terms in the original Tsodyks-Markram model by adding a tangent function to prevent tonic endless spiking. Such tonic spiking was observed when the network fired in high rates and originated from the linearity between the recovery rate and the amount of inactive resources. Our modification is in accordance with findings indicating that synapses do not increase their recovery rate following depletion, but rather decrease it after a certain level of depletion [71–73].

The synaptic input to a neuron, *I*_*syn*_, was calculated by summing over synaptic currents from all connected neurons: $I_{syn}(t)=\sum_{i}A_{i}y_{i}(t)$

where *A*_*i*_ is the synaptic strength. To reflect the non-uniformity of synaptic strengths, they were selected from a Gaussian distribution (*μ* = *A*_*nom*_, *σ* = *A*_*nom*_/2), where *A*_*nom*_ is the nominal value of the synapse [70,74] (see Table 2 for a full list). To limit the distribution of strengths, only values between 0.8*A*_*nom*_ ≤ *A* ≤ 1.2*A*_*nom*_ were considered (values were redrawn from the distribution until a value within limits was drawn).

## Supporting Information

S1 FigCell number distributions in clusters.(A) A distribution of the estimated number of cells for different clusters (see main text). (B) The ratio between the number of cells in the bigger cluster and the smaller cluster in cluster pairs. (C) The average delay between the sending cluster and the receiving cluster (see Fig 3E) as a function of the number of cells in the receiving cluster. (D) The asymmetry in cluster activity (see Fig 4D) as a function of the normalized difference in cell numbers: $\frac{2(N1-N2)}{N1+N2}$, where N1 and N2 are the number of cells in the sending and receiving clusters respectively. In total, 88 cluster pairs were analyzed (corresponding to 176 data points in (C)). One cluster pair with one very large cluster (~1700 cells) was removed to not bias the results.(TIF)

S2 Fig NBs represent synchronized multi-unit firing. (A) Bright field image of a cluster grown on an high density MEA (30 μm distance between electrode centers, 10 μm electrode diameter). (B) Voltage traces recorded from the cluster in (A) during a network burst (traces of broken channels were removed). (C) Zoom into Voltage traces in (B) for a subset of ten electrodes (red dots in (B)). The differences in the temporal structure of multi-unit activity suggest that many neurons in the cluster are synchronously activated during this network event (similar variability between different electrodes was observed during 100 consecutive network events recorded from this cluster and for two other clusters recorded with high-density MEAs).
(TIF)

S3 Fig Recruitment time in clusters. (A) Normalized AI of 500 consecutive NBs from one cluster aligned by the NB peak (see NB detection in Materials and Methods). (B) The distribution of the time to peak (peak times minus start times) for all NBs from the cluster in (A). (C) The average AI over all NBs in (A). The red lines in (B) and (C) show the mean calculated recruitment time. (D) A distribution of the mean recruitment time for the same population of cluster pairs as in Fig 3.
(TIF)

S4 FigBi-modality in cluster responses.(A) Normalized AI in the receiving cluster as a function of AI in the sending cluster for consecutive NB from the cluster pair analyzed in Fig 3D (first 1000 NBs are shown). (B) The distribution of data points in (A) projected on the diagonal (red line in (A)). The bi-modality of the distribution is quantified by the Bimodality coefficient ($BC=\frac{{m_{3}}^{2}+1}{m_{4}+3\frac{{\left(n-1\right)}^{2}}{\left(n-2)(n-3\right)}}$, where m_3_ is the skewness of the distribution, m_4_ is the kurtosis and n is the number of samples used for estimation). (C) Distribution of BC over all the cluster pairs analyzed in Fig 3.(TIF)

S5 Fig Differences in NB sizes for uniform and clustered networks. (A) Distribution of the normalized number of active electrodes during 1000 consecutive NBs from 15 clustered networks (different networks are color coded). (B) Distribution of the normalized bounding area for the NBs in (A). The bounding area is defined as the minimal rectangular area that included all active electrodes during an NB and represents the spatial spread of the NB. (C) and (D) are the same as (A) and (B) respectively, but for eight uniform networks. In all plots, the measured parameter was normalized to the maximal value in all NBs in each network to enable a common metric for all networks. The distribution for each network was calculated on this normalized parameter (to give the same weight for every network) and divided by the number of networks in the final stack histogram. The total height of the stack represents the population average over all networks.
(TIF)
