# Supplementary figures and images for: Modularity Induced Gating and Delays in Neuronal Networks
Source: PLoS Comput Biol. 2016 Apr 22;12(4):e1004883. doi: 10.1371/journal.pcbi.1004883 (PMC4841573; doi:10.1371/journal.pcbi.1004883)

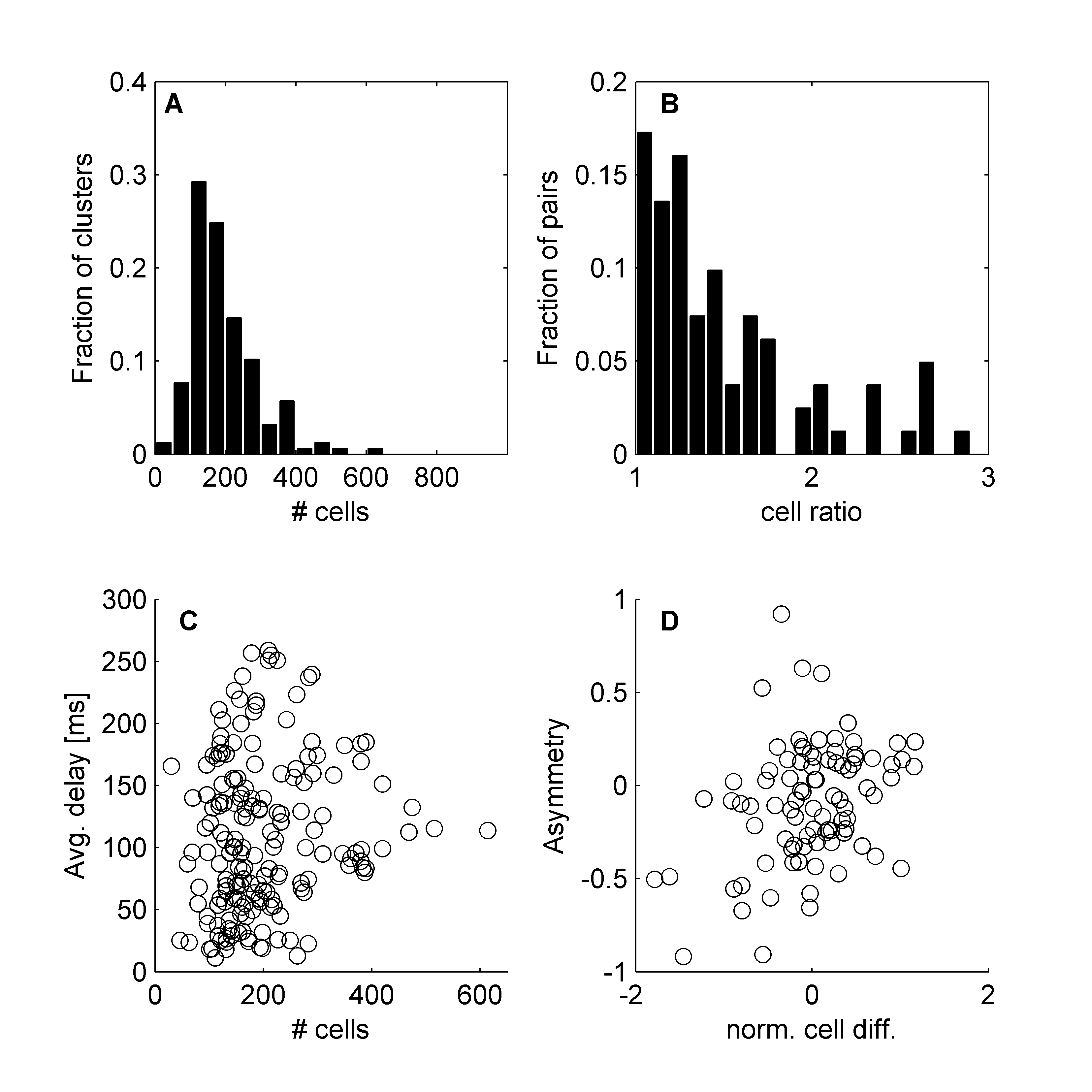

Supplement: S1 Fig — (A) A distribution of the estimated number of cells for different clusters (see main text). (B) The ratio between the number of cells in the bigger cluster and the smaller cluster in cluster pairs. (C) The average delay between the sending cluster and the receiving cluster (see Fig 3E) as a function of the number of cells in the receiving cluster. (D) The asymmetry in cluster activity (see Fig 4D) as a function of the normalized difference in cell numbers: 2(N1−N2)N1+N2, where N1 and N2 are the number of cells in the sending and receiving clusters respectively. In total, 88 cluster pairs were analyzed (corresponding to 176 data points in (C)). One cluster pair with one very large cluster (~1700 cells) was removed to not bias the results. (TIF) [file pcbi.1004883.s001.tif]

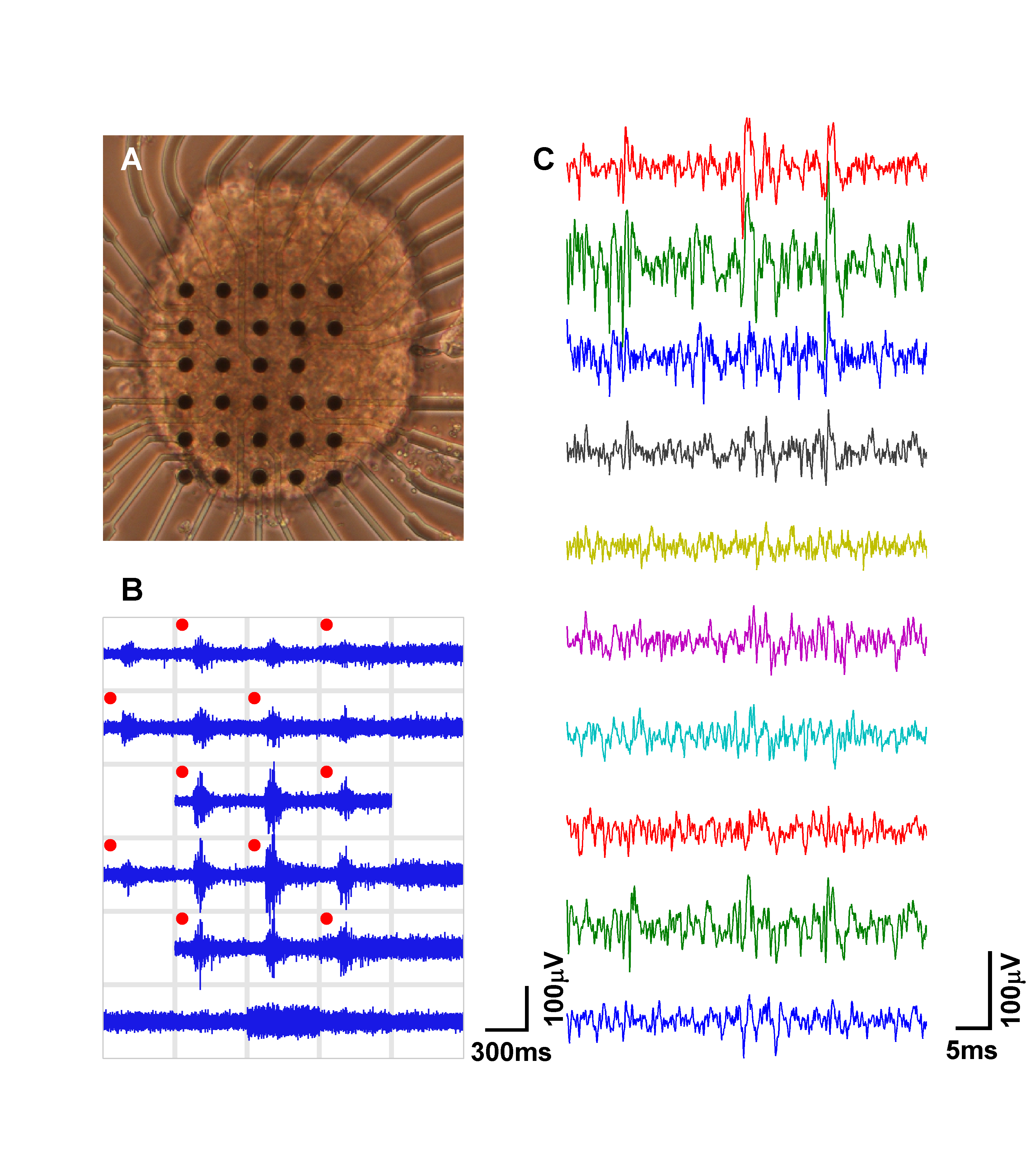

Supplement: S2 Fig — (A) Bright field image of a cluster grown on an high density MEA (30 μm distance between electrode centers, 10 μm electrode diameter). (B) Voltage traces recorded from the cluster in (A) during a network burst (traces of broken channels were removed). (C) Zoom into Voltage traces in (B) for a subset of ten electrodes (red dots in (B)). The differences in the temporal structure of multi-unit activity suggest that many neurons in the cluster are synchronously activated during this network event (similar variability between different electrodes was observed during 100 consecutive network events recorded from this cluster and for two other clusters recorded with high-density MEAs). (TIF) [file pcbi.1004883.s002.tif]

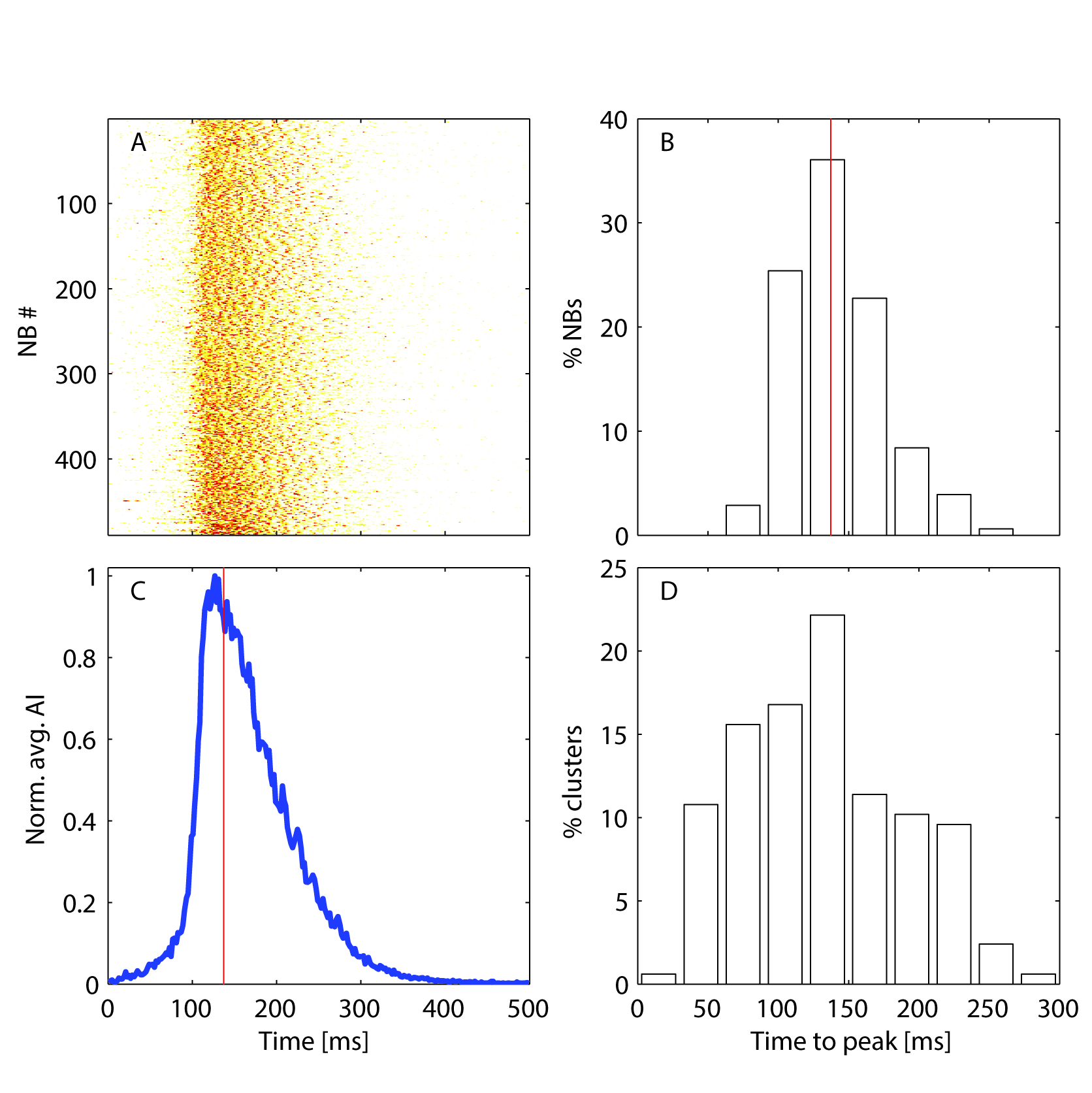

Supplement: S3 Fig — (A) Normalized AI of 500 consecutive NBs from one cluster aligned by the NB peak (see NB detection in Materials and Methods). (B) The distribution of the time to peak (peak times minus start times) for all NBs from the cluster in (A). (C) The average AI over all NBs in (A). The red lines in (B) and (C) show the mean calculated recruitment time. (D) A distribution of the mean recruitment time for the same population of cluster pairs as in Fig 3. (TIF) [file pcbi.1004883.s003.tif]

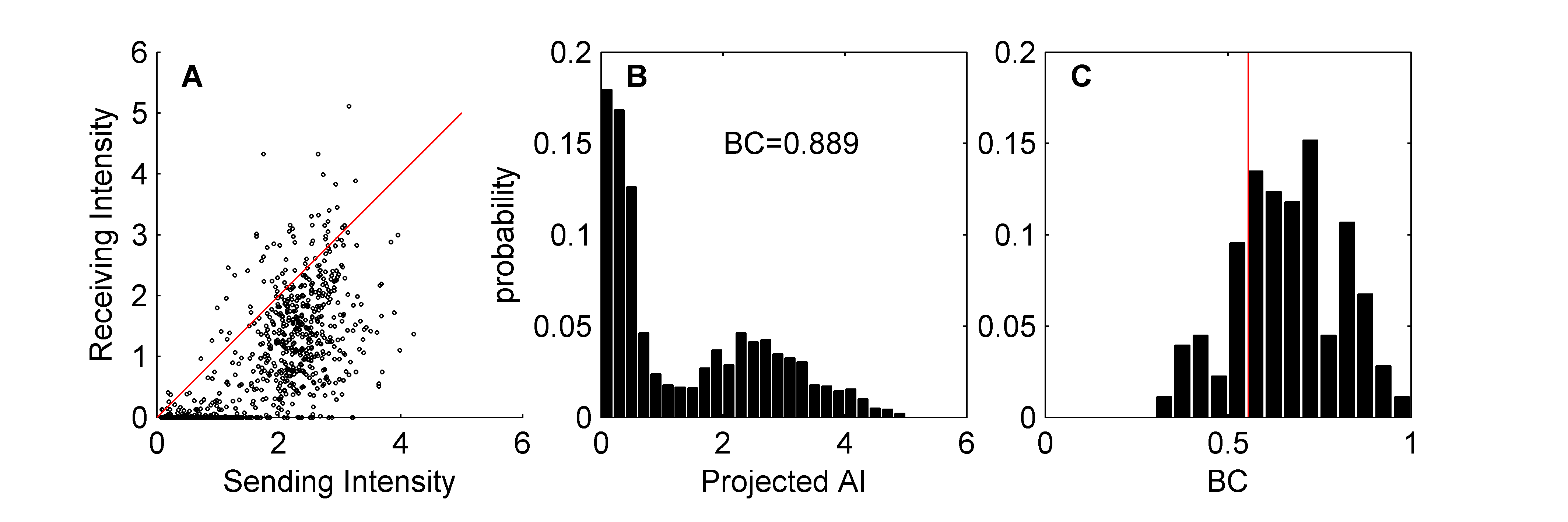

Supplement: S4 Fig — (A) Normalized AI in the receiving cluster as a function of AI in the sending cluster for consecutive NB from the cluster pair analyzed in Fig 3D (first 1000 NBs are shown). (B) The distribution of data points in (A) projected on the diagonal (red line in (A)). The bi-modality of the distribution is quantified by the Bimodality coefficient (BC=m32+1m4+3(n−1)2(n−2)(n−3), where m3 is the skewness of the distribution, m4 is the kurtosis and n is the number of samples used for estimation). (C) Distribution of BC over all the cluster pairs analyzed in Fig 3. (TIF) [file pcbi.1004883.s004.tif]

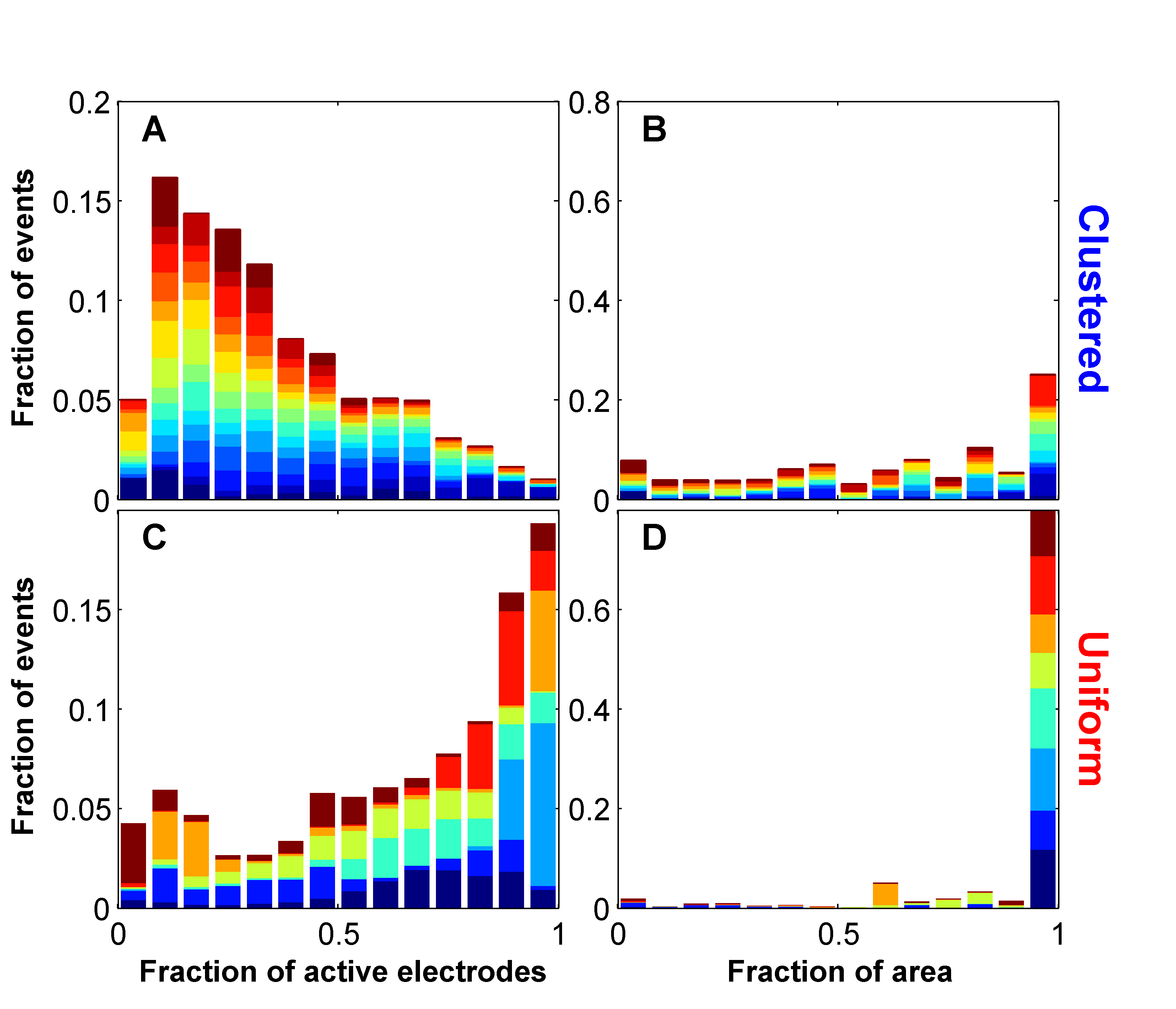

Supplement: S5 Fig — (A) Distribution of the normalized number of active electrodes during 1000 consecutive NBs from 15 clustered networks (different networks are color coded). (B) Distribution of the normalized bounding area for the NBs in (A). The bounding area is defined as the minimal rectangular area that included all active electrodes during an NB and represents the spatial spread of the NB. (C) and (D) are the same as (A) and (B) respectively, but for eight uniform networks. In all plots, the measured parameter was normalized to the maximal value in all NBs in each network to enable a common metric for all networks. The distribution for each network was calculated on this normalized parameter (to give the same weight for every network) and divided by the number of networks in the final stack histogram. The total height of the stack represents the population average over all networks. (TIF) [file pcbi.1004883.s005.tif]
